# Breaking the blue barrier of nucleobase fluorescence emission with dicyanovinyl-based uracil molecular rotor probes†

**DOI:** 10.1039/d4ra07000c

**Published:** 2024-11-25

**Authors:** Mria Chowdhury, Akym John, Robert H. E. Hudson

**Affiliations:** a Department of Chemistry, Western University London Ontario N6A 5B7 Canada rhhudson@uwo.ca

## Abstract

Dicyanovinyl-modified uracil produces fluorescent molecular rotors (FMR) that display massively red-shifted emission and huge Stokes shifts. They are exemplified by DCVSU – an intrinsically fluorescent nucleobase analog (IFNA) with the longest emission wavelength of 592 nm (DMSO) reported thus far which also shows strong polarity sensitivity and large Stokes shift (*λ* = 181 nm). The IFNAs exhibited typical molecular rotor response to solvent viscosity with brightnesses (*ε* × *φ*) of up to 8700 cm^−1^ M^−1^. ^1^H NMR titration confirmed the expected association of the IFNA with the complementary nucleobase adenine-9-ethyl acetate.

## Abstract 2

Dicyanovinyl-modified uracil produces fluorescent molecular rotors (FMR) that display massively red-shifted emission and huge Stokes shifts.

## Graphical abstract

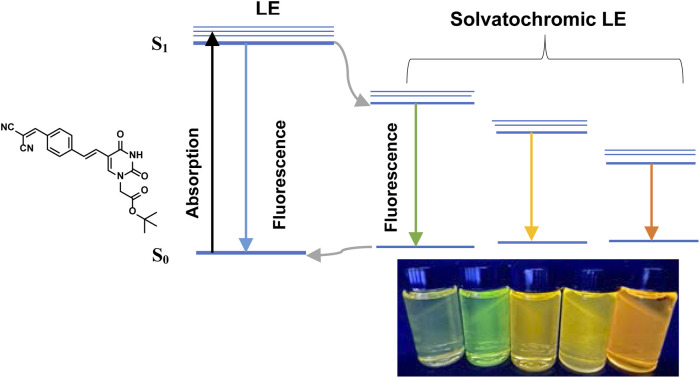

## Introduction

The search for intrinsically fluorescent nucleobase analogs (IFNAs) that are environmentally sensitive, have comparatively red-shifted emission wavelengths and are bright continues since such analogs can aid the efficient detection of nucleic acid structures, dynamics and functions.^1–3^ Standard fluorescent tags^4^ tethered to nucleosides for use in diagnostic assays are limited in their detection ability such as sensitivity towards hybridization with complementary strands, detection of abasic sites and gene mutations.^5^ Covalent modification of nucleobases such that they are integrated into an autofluorescent moiety while retaining base-pairing ability has the potential to be exploited as a diagnostic and research tool.^5,6^

Fluorescent molecular rotor (FMR) probes have gained increased attention from researchers in recent years and are extensively used in biosensing applications.^7–10^ Structurally, FMRs typically have electron donor and acceptor moieties separated by a rotatable linker resulting in microenvironmental sensitivity to viscosity,^11^ hybridization,^12^ polarity, and pH.^13^ This provides useful indicators of changes within cells and organelles, protein aggregates, or interactions between DNA–protein or aptamers, polymers and other complex molecular environments.^14–16^ Intrinsically modified nucleobase FMRs so far fall short in addressing some of the above-mentioned requirements such as insufficient red-shifted emission or low brightness precluding effective use in biological assays.

**Fig. 1:**
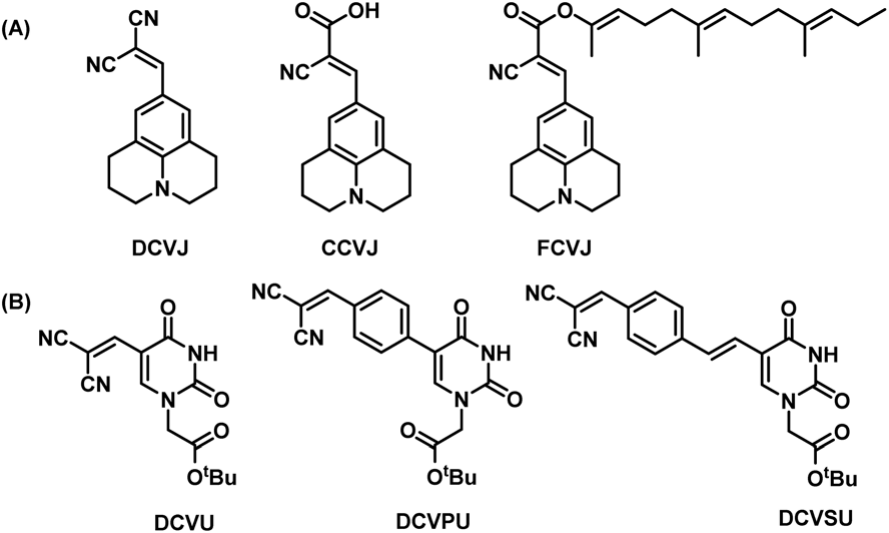
(A) Structures of DCVJ, CCVJ, FCVJ; (B) structures of chimeric nucleobase FMR synthesized for this study DCVU, DCVPU, DCVSU.

Previously, we reported the “chimeric strategy”^17^ that was employed to guide the design of chemically modified nucleobases such that they would possess fluorescence or quenching properties yet retaining their hybridizing ability with complementary nucleobases. In our endeavour to ‘break the blue barrier’ common to most IFNAs and produce red-shifted emission to overcome interference from background auto-fluorescence of the biological milieu, we have taken the chimeric approach of linking part of a well-known fluorescence rotor, dicyanovinyljulolidine (DCVJ, Fig. 1A)^18^ to uracil nucleobase. The design retains the dicyanovinyl group as the acceptor and replaces the donor julolidine group with uracil (DCVU, Fig. 1B). Previously, the 5-dicyanovinyluracil-based nucleoside analogue was screened for anti-Leishmania activity; however, the photophysical properties were not reported.^19^ While DCVJ and its analogues like CCVJ (9-(2-carboxy-2-cyanovinyl)julolidine) and FCVJ ((2-carboxy-2-cyanovinyl)-julolidine farnesyl ester) are popularly used FMR dyes used^20–22^ (Fig. 1A), CCVJ inspired analogues have been tethered to cytosine nucleobase^12^ and the resulting photophysical properties were not much different from CCVJ itself.

Additional structural modifications to influence the fluorescence properties of dicyanovinyl uracil molecular rotors were made by introducing phenyl or styryl linking groups in tandem with the dicyanovinyl (DCV) group. The DCVJ-inspired uracil modifications (Fig. 1B) reported herein have dramatically different emission wavelengths and Stokes shifts as compared to DCVJ and CCVJ.

## Results and discussion

Pd-catalyzed coupling reactions are an effective way to introduce various substituents at the C5 position of uracil nucleobase. Suzuki–Miyaura coupling reaction between different boronic acids and 5-halo-uracil/uridine derivatives in the presence of Pd catalyst has been used in the synthesis of a variety of functionalized uracil analogues.^23,24^ For the present work, uracil was first converted to 5-iodouracil^25^ followed by regioselective alkylation at the N^1^ position to yield 1 (see ESI: Scheme SI-1†). Compound 1 served as the coupling partner with commercially available 4-formylphenylboronic acid under Suzuki–Miyaura reaction conditions to yield 2b, full details are given in the ESI.† Alternatively, 1 was reacted with (*E*)-(4-formylstyryl)boronic acid (Scheme SI-2†) under the same coupling conditions to give 2c. (*E*)-(4-Formylstyryl)boronic acid was synthesized using Heck coupling reaction between *p*-bromobenzaldehyde and vinyl-boronic acid pinacol ester. When carried out at an elevated temperature (90 °C) for 48 h, the Heck reaction gave an improved yield of 72% in our hands over the literature procedure (Scheme SI-2†).^26^

**Scheme 1:**
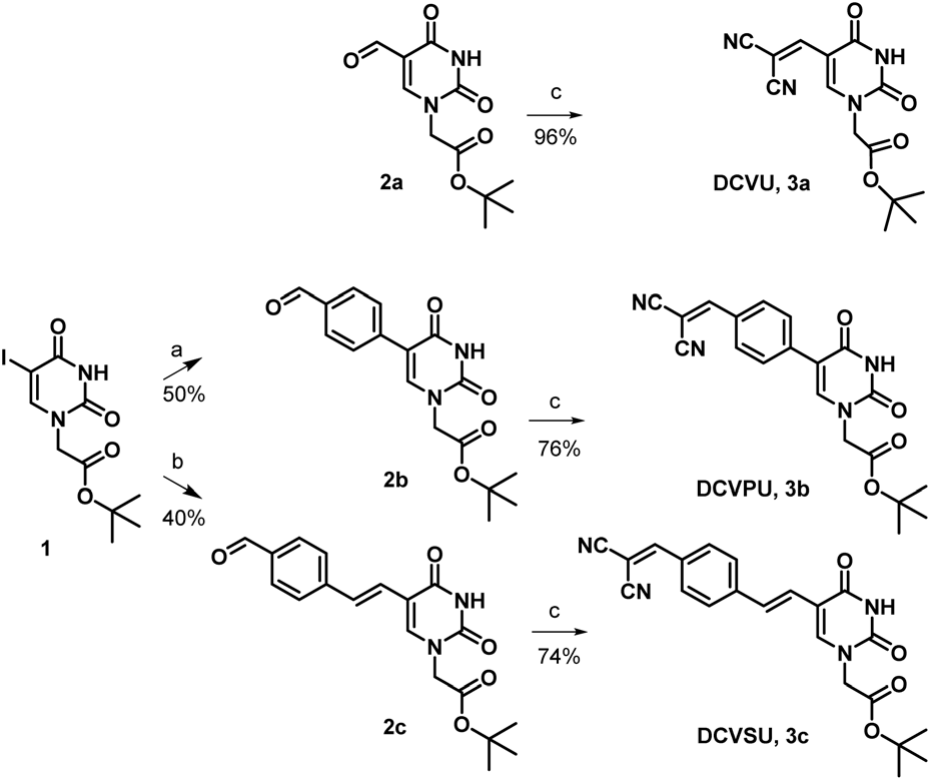
Reagents and conditions (a) 4-formylphenylboronic acid, K_2_CO_3_, Pd(dppf)Cl_2_, THF/H_2_O (4 : 1), 100 °C, 5 h, (b) (4-formylstyryl)boronic acid, K_2_CO_3_, Pd(dppf)Cl_2_ THF/H_2_O (4 : 1), 100 °C, 5 h, (c) malononitrile, EtOH, 50 °C, 16 h.

Linker-less attachment of the dicyanvinyl group to uracil was achieved using substrate 2a which was synthesized from uracil *via* 5-hydroxymethyl uracil followed by oxidation according to our previously reported method.^17^ The dicyanovinyl-functional group was introduced by reaction of the precursor aldehydes 2a–c with malononitrile in ethanol at 50 °C (stirred, overnight) in a Knoevenagel-type reaction without the presence of an exogenous base. In this manner, the DCV-based uracil nucleobase fluorophores 3a (DCVU), 3b (DCVPU), and 3c (DCVSU) were achieved (Scheme 1).

**Fig. 2:**
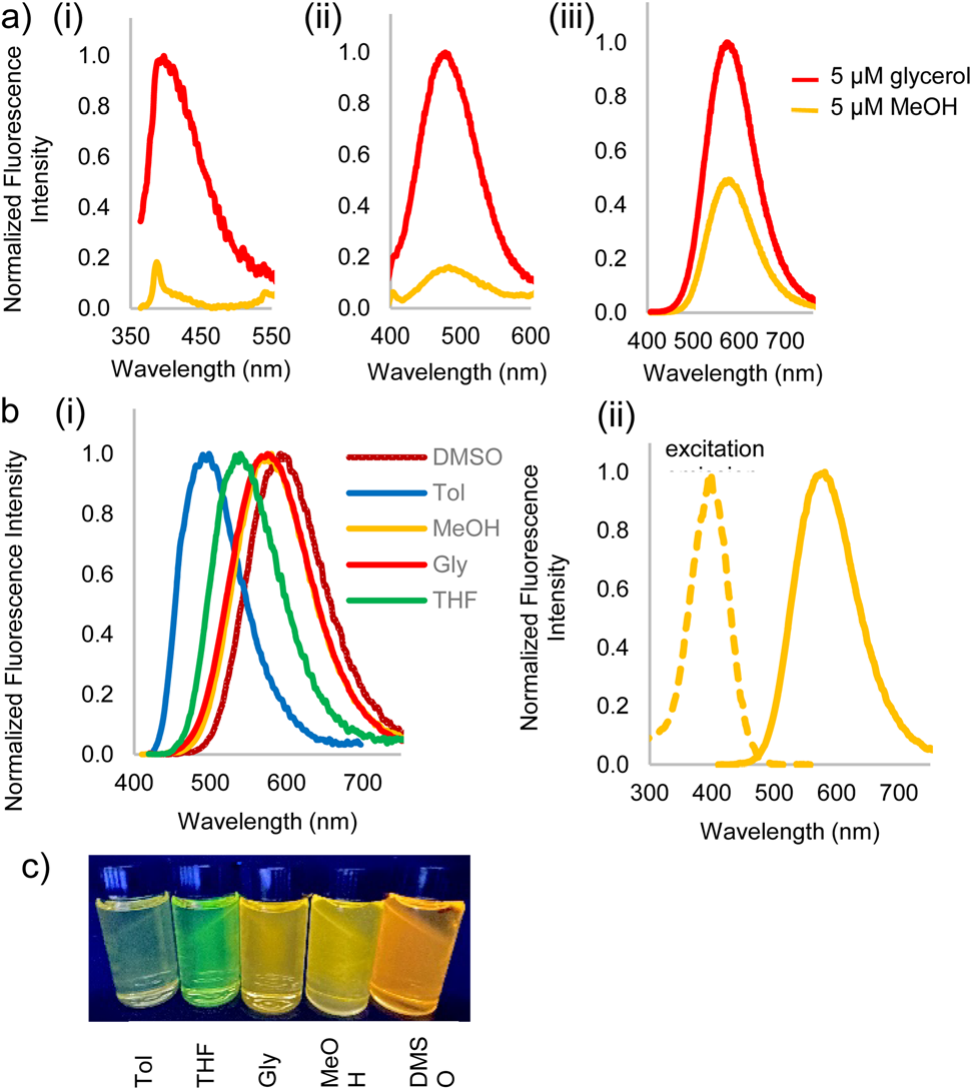
(a) Fluorescence emission spectra in 5 μM solutions of glycerol (with 5% MeOH) and methanol (MeOH) to study viscosity dependence of (i) DCVU 3a, (ii) DCVPU 3b, (iii) DCVSU 3c, (b) (i) emission spectra of 5 μM 3c in DMSO, glycerol, methanol, THF, toluene showing polarity dependence [*λ*_ex_(DMSO) = 592 nm, *λ*_ex_(glycerol) = 573 nm, *λ*_ex_(methanol) = 575 nm, *λ*_ex_(THF) = 537 nm, *λ*_ex_(toluene) = 496 nm], (ii) excitation and emission spectra of 5 μM 3c in methanol showing large Stokes shift, (c) picture of 3c in different solvents: from left-to-right: Tol (toluene), THF (tetrahydrofuran), Gly (glycerol), MeOH (methanol), DMSO (dimethylsulfoxide).

Fluorophores 3a–c and 2c were then subjected to photophysical characterization, Fig. 2 and ESI.† UV-Vis spectroscopy and fluorescence measurements were done for compounds 3a–c and 2c in solvents of varying viscosities and polarities. Data for DCVSU (3c) is shown in Table 1. Complete characterization for 3a–c and 2c is provided in SI-Table 1.†

**Table 1:** Photophysical properties of DCVSU (3c)

Property	Solvent				
	DMSO	Gly^a^	MeOH	Tol^a^	THF
*λ*_abs,max_ (nm)	411	400	395	402	404
*λ*_em,max_ (nm)	592	573	575	496	537
Δ*λ* (nm) (Δ*ν* 10^3^ cm^−1^)	181 (7.4)	173 (7.5)	180 (7.9)	94 (4.7)	133 (6.1)
*ε* (10^4^ cm^−1^ M^−1^)	4.2	3.7	2.7	3.1	2.4
*φ*	0.21	0.22	0.09	0.01	0.05
*ε* × *φ* (cm^−1^ M^−1^)	8700	8000	2390	310	1190

a Gly = glycerol, Tol = toluene, Δ*ν* = Stokes shift in wavenumbers, *ε* = extinction coefficient, *φ* = quantum yield, *ε* × *φ* = brightness factor.

As conjugation increases from DCVU to DCVSU, the emission wavelengths undergo a bathochromic shift by approximately 100 nm, going from 389 nm (DMSO) for DCVU to 503 nm (DMSO) for DCVPU to 592 nm (DMSO) for DCVSU, Fig. 2. Such a trend was observed for all the solvents used in this study. To the best of our knowledge, the emission maxima of 573 nm (glycerol), 575 nm (methanol) and 592 nm (DMSO) are the most red-shifted fluorophores reported for any nucleobase FMR or any other intrinsically modified nucleobase fluorophores^23^ known to date; even more red-shifted than a recently reported “most red-shifted” non-FMR push–pull C-linked 8-(diethylamino)benzo[*b*][1,8]naphthyridin-2(1*H*)-one nucleoside (ABN).^27^

The brightness for DCVSU is nearly as great as some of the recently reported bright modified nucleobase fluorophores^27,28^ (*ε* = 2.0–4.0 × 10^4^ measured at the absorption maxima in different solvents, with the highest quantum yield (*φ*) of 0.21 and 0.22 in DMSO and glycerol, respectively). Quantum yields were measured using fluorescein or rhodamine as standards.

Fluorescence turn-on, ascribed to the molecular rotor behaviour, was observed in a more viscous solvent (glycerol) and low fluorescence (3b, 3c) to complete fluorescence turn-off (3a) was observed in a less viscous solvent (methanol). Isomerization of the pseudo *cis*–*trans* of C=C bond in dicyanovinyl group in the excited state is likely responsible as the main non-radiative deactivation channel from S_1_ (excited state) to S_0_ (ground state), as reported for DCVJ.^29^ While DCVU (3a) shows complete fluorescence turn-off in a non-viscous solvent, increasing the conjugation length through DCVPU to DCVSU results in a less dramatic turn-off of fluorescence, Fig. 2a. Increased conjugation increases the barrier of rotation of C=C in S_1_ state, thus increased fluorescence is observed and decrease in non-radiative decay.

**Fig. 3:**
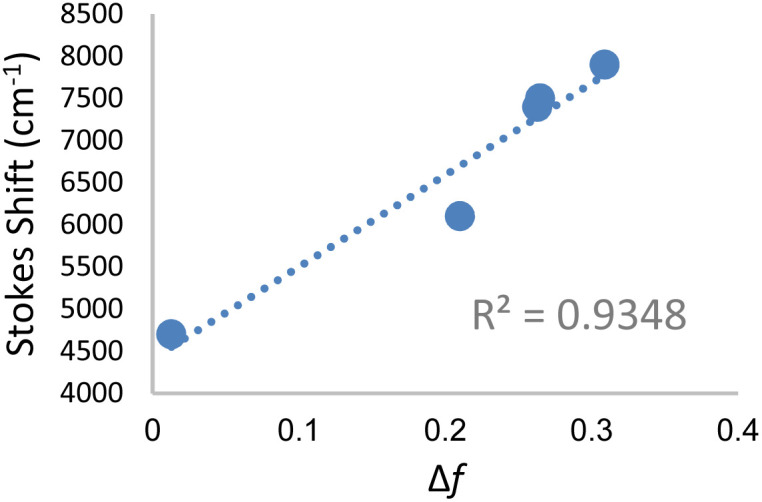
Lippert–Mataga plot for DCVSU (3c) in solvents toluene, THF, methanol, glycerol and DMSO.

Significant polarity-dependent solvatochromism was also observed and 100 nm change covering the range from blue to orange region of the visible spectrum was observed, Fig. 2b. Polarity induced solvatochromism can be quantified by Lippert–Mataga plot which draws a relationship between Stokes shift (Δ*ν̄* in wavenumber) and orientation polarizability Δ*f* (eqn (1a) and (1b)) derived by Lippert and Mataga.^30,31^1a
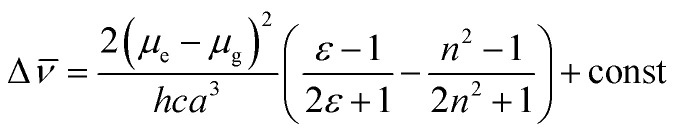
1b
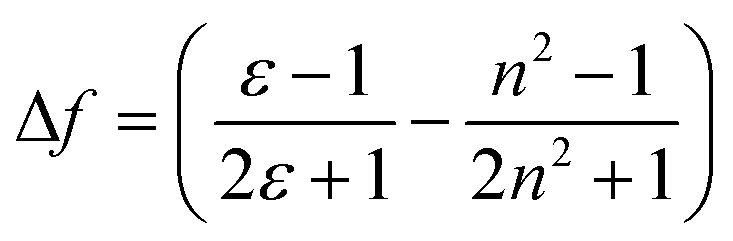
where, *μ*_e_ and *μ*_g_ are excited and ground-state dipole moments, *c* is the velocity of light, *h* is the Planck's constant, and “*a*” is the Onsager cavity radius of the fluorophore. A positive slope, as obtained for 3c when plotted for solvents of different polarities (Fig. 3), is indicative of increased dipole moment in the excited state than in the ground state, thus giving rise to significant solvent-dependent shifts in emission spectra. We have also observed similar linear graph with positive slope when we plotted Stokes shift *vs.* Reichardt's *E*_T_(30).^32^ The bathochromic shift of emission wavelengths with an increase in solvent polarity is rationalized on the ability of polar solvents stabilize the polarized donor–acceptor character of the fluorophore in the excited state. The large Stokes shift >150 nm in almost all the solvents for DCVSU, specifically 181 nm for DCVSU in DMSO (Fig. 2c and SI-1†) is higher than commonly used fluorescent probes and the highest among intrinsic nucleobase fluorophores. Fluorophores with large Stokes shifts are desirable because there is a reduction in self-quenching and has applications in fluorescence imaging of biological assays including the ability to expand colour multiplexing and decrease signal-to-noise ratio in fluorescence microscopy.^33–35^

According to the conventional description of FMRs, rotors with more donor–acceptor characteristics show more intramolecular charge transfer (ICT) and exhibit polarity sensitivity and not viscosity sensitivity and *vice versa* for rotors with less ICT behaviour, although deviations from this trend have been observed previously with some dicyanovinyl-based rotors by Zhou *et al.*^29^ Similarly, 3a–c exhibit both polarity and viscosity sensitivity making them more environmentally sensitive, as compared to DCVJ.^18^ Like the archetypal FMR DCVJ, we hypothesize that fluorescence emission of 3a–c occurs from the LE (locally excited) state and not the lower energy TICT (twisted intramolecular charge transfer) state which is dark.^36^ This is evident from the similarity of emission wavelengths in the low-viscosity solvent methanol and viscous solvent glycerol. If TICT was not dark, then we would see LE emission for modified nucleobase in glycerol and TICT emission for methanol giving us different emission wavelengths. Methanol and glycerol have dramatically different viscosities but similar relative polarity (methanol = 0.76 and glycerol = 0.79); thus, it was deduced that emission was from the LE. Additionally, in DMSO, 3c has a relatively high quantum yield of emission (*φ* = 0.21), which is possible when the emission is from LE since the increase in the TICT population leads to increased non-radiative decay.

**Fig. 4:**
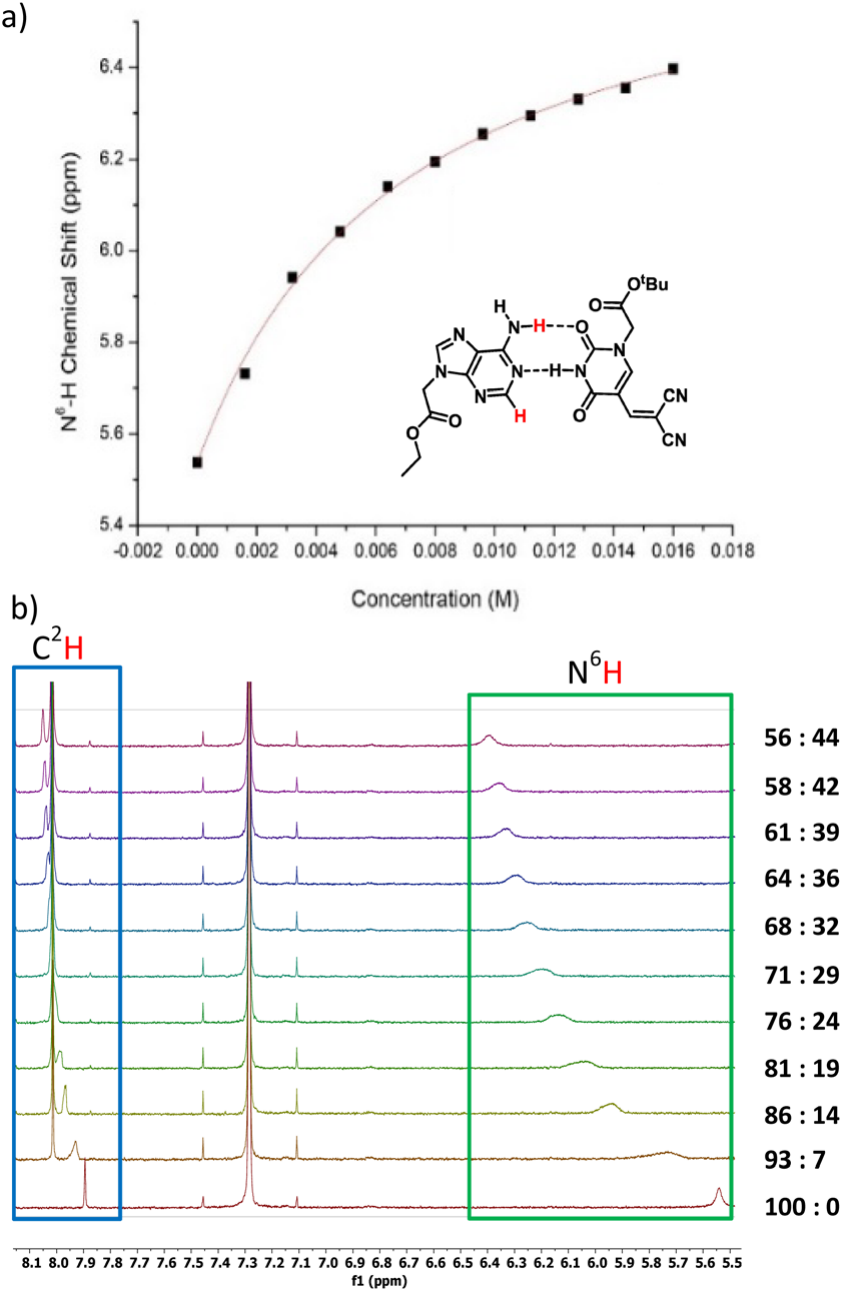
(a) N^6^H chemical shift of adenine (host) *vs.* concentration of 3a (guest) plot using non-linear curve fit. Watson–Crick base pair between adenine-9-ethyl acetate and modified uracil 3a, (b) ^1^H NMR titration of 0.5 mM adenine-9-ethyl acetate (A = host) with 20 mM 3a (guest). A : DCVU represents volume ratios of A and 3a on successive additions of 3a without changing the concentration of A.

Next, we investigated the modified nucleobases for their ability to base pair with the complementary nucleobase adenine (Fig. 4a). Anhydrous CDCl_3_ was used to perform titration to measure the association of 3a and adenine-9-ethyl acetate by ^1^H NMR spectroscopy. This pair was chosen because the base-pairing faces of 3a–c are identical and 3a has better solubility in CDCl_3_ than 3b or 3c. When 0.5 mM adenine-9-ethyl acetate (host) was titrated with 20 mM of 3a, the association was evident from the downfield shifting of N^6^H proton of adenine-9-ethyl acetate from 5.54 to 6.40 ppm due to H-bonding interactions. Concomitantly, the C^2^H peak also shifted downfield from 7.89 to 8.05 ppm owing to pseudo-H bonding^37^ interactions (Fig. 4b). An association constant, *K*_a_ of 155 ± 10 M^−1^ for N^6^H⋯O H-bonding (Fig. 4a) and 155 ± 13 M^−1^ for C^2^H⋯O interactions (Fig. SI-5†) were calculated. The *K*_a_ values were within the range of values observed for 5-substituted uracil and adenine previously in the literature.^38,39^ The slightly higher association constant compared to A:T may be attributed to the electron-withdrawing nature of the dicyanovinyl group in the C^5^ position of modified uracil 3a, which is expected to have an acid-strengthening effect on the N^3^H and consequently increase in H-bonding ability.^40^

## Conclusions

We have reported a series of new dicyanovinyl-modified uracil fluorescent nucleobases that exhibited environmentally sensitive fluorescence emissions from the blue to yellow/orange region of the visible spectrum. Such IFNAs have environmental sensitivity which may translate to applications for reporting interactions of nucleic acids with biomolecules such as complementary nucleic acids or proteins. The large Stokes shift and competitive brightness as compared to some of the brightest but “blue” emitting IFNAs recently developed makes these IFNAs great candidates for applications in fluorescence microscopy. The ease of synthesis combined with exceptional fluorescence properties make DCVSU (3c) a much-needed addition to the IFNA FMR toolbox.

With evidence of strong binding constant, *K*_a_, research on these chimeric IFNAs is being expanded to incorporate them into peptide nucleic acid (PNA) strands in our laboratories to assess the sequence dependency, hybridization with complementary strands and effect of hybridization on fluorescence.

## Data availability

The data supporting this article have been included as part of the ESI.†

## Author contributions

Funding acquisition, conceptualization, supervision and writing – review & editing (RHEH); investigation, visualization and writing – original draft (MC); investigation (AJ).

## Conflicts of interest

There are no conflicts to declare.

## Supplementary Material

RA-014-D4RA07000C-s001
