# Supplementary material for: Breaking the blue barrier of nucleobase fluorescence emission with dicyanovinyl-based uracil molecular rotor probes
Source: RSC Adv. 2024 Nov 25;14(50):37605–9. doi: 10.1039/d4ra07000c (PMC11586925; doi:10.1039/d4ra07000c)
Supplement: RA-014-D4RA07000C-s001 [file RA-014-D4RA07000C-s001.pdf]

Supplementary Information (SI) for RSC Advances.
This journal is © The Royal Society of Chemistry 2024

BREAKING THE BLUE BARRIER OF NUCLEOBASE FLUORESCENCE EMISSION WITH DICYANOVINYL-BASED URACIL MOLECULAR ROTOR PROBES

Mria Chowdhury,[1] Akym John,[1] and Robert H.E. Hudson[1,*]

[1]Department of Chemistry, The University of Western Ontario, London, Ontario, Canada N6A 5B7.

Corresponding Author - Robert H.E. Hudson (rhhudson@uwo.ca)

TABLE OF CONTENTS

1. Syntheses ....SI 3 – SI 7
2. Photophysical properties ....SI 8 – SI 11
3. Lippert-Mataga plot for polarity dependent solvatochromism ....SI 12
4. Complementary nucleobase association studied by ^{1}H NMR.....................................SI 14 – SI 16
5. NMR spectra ....SI 17 – SI 25
6. ESI-MS data ....SI 26 – SI 27
7. References ....SI 28

SYNTHESIS

All chemicals were obtained from commercial sources and were of ACS reagent grade or higher and were used without further purification. Anhydrous and HPLC-grade solvents for PNA synthesis and chromatography were purchased from Caledon Laboratories. All other solvents were dried by passing through activated alumina columns. In all cases, sodium sulfate was used as the drying agent and solvent was removed by reduced pressure with Buchi Rotavapor. Thin-layer chromatography was performed on Silicycle Silica Gel TLC F-254 plates. Unless otherwise specified the Rf values are reported in the solvent system the reaction was monitored in. Flash chromatography was performed with Silicycle SiliaFlash® F60 230-400 mesh silica. All chemical shifts are reported in parts per million (δ), from tetramethylsilane (0 ppm), and are referenced to the residual proton in the respective solvent: $CDCl_3$ (7.26 ppm) and DMSO-d6 (2.50 ppm) for ^{1}H NMR and $CDCl_3$ (77.0 ppm) and DMSO-d6 (39.5 ppm) for ^{13}C NMR. Multiplicities are described as s (singlet), d (doublet), t (triplet), q (quartet), m (multiplet) and br s (broad singlet. Spectra were obtained on Bruker-400 and INOVA-400 and INOVA-600 instruments. High-resolution mass spectra (HRMS) were obtained using electrospray ionization (ESI) and time-of-flight (TOF) mass analyzer.

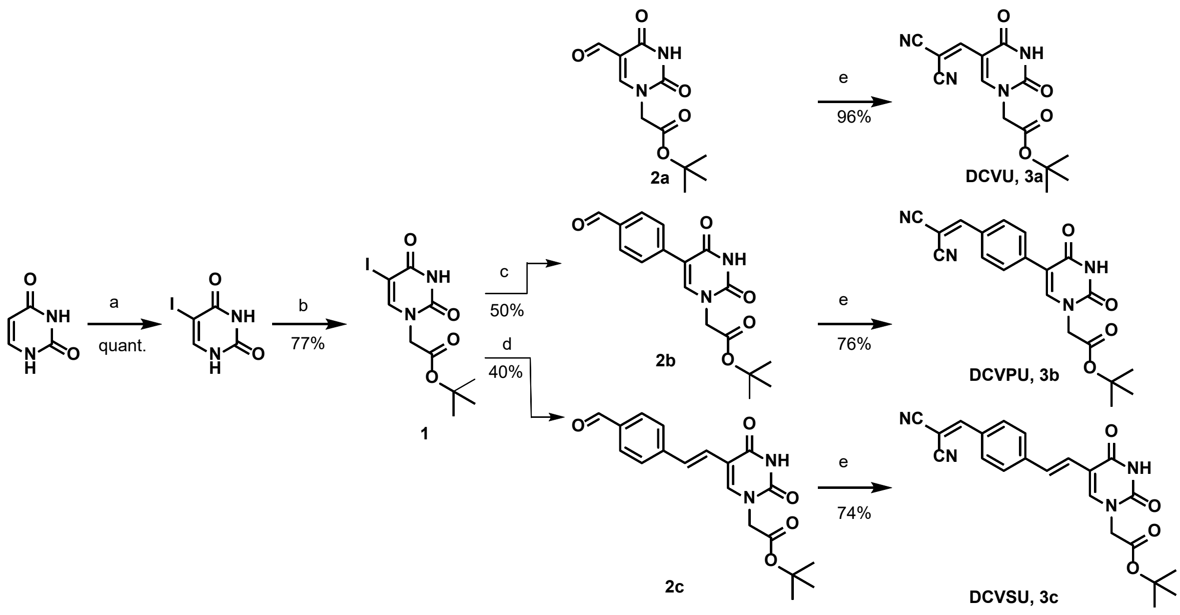

Scheme SI-1. Reagents and conditions a) ICl, MeOH, 50 °C, 4 h, b) TEA, *t*-butylbromoacetate, dry DMF, rt. 24 h, c) 4-formylphenylboronic acid, K_2CO_3, $Pd(dppf)Cl_2$, THF/H_2O (4:1), 100 °C, 5 h, d) **SI-2**, K_2CO_3, $Pd(dppf)Cl_2$, THF/H_2O (4:1), 100 °C, 5 h, e) malononitrile, EtOH, 50 °C, 16 h.

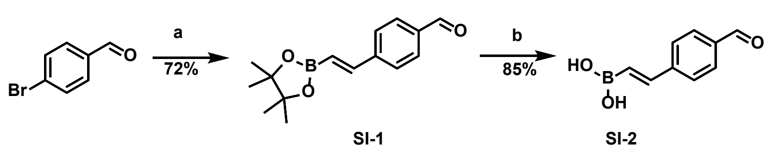

Scheme SI-2. Reagents and conditions a) Vinylboronic acid pinacol ester, $Pd(OAc)_2$, 1,10-phenanthroline, Et_3N, ACN, 90 °C, 48 h, b) i. $NaIO_4$, THF/H_2O, 30 min, rt, ii. 1 M HCl, 2 h, rt.

5-Iodouracil

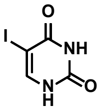

Uracil (1.43 g, 12.8 mmol) was suspended in dry methanol (55 mL) under nitrogen gas. Iodine monochloride (4.13 g, 25.4 mmol) was added to the solution and the mixture heated at 50 °C for 4 h. After completion, the reaction was cooled to room temperature, filtered and the ppt. washed with diethyl ether. 1.8 g (quantitative) of white powder was obtained. ^{1}H NMR (400 MHz, DMSO-d6) δ 11.39 (s, 1H), 11.15 (s, 1H), 7.88 (s, 1H). ^{13}C NMR (100.6 MHz, DMSO-d6) δ 161.5, 151.2, 146.9, 67.6.[2]

tert-Butyl 2-(uracil-5-iodo-1-yl)acetate (**1**)

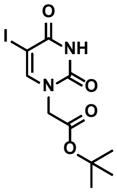

5-iodouracil (2.5 g, 17.8 mmol) was suspended in 50 mL of dry DMF at room temperature. Triethylamine (2.5 mL, 17.8 mmol) was added to the solution. *tert*-Butyl bromoacetate (3.1 mL, 21.4 mmol) was added drop wise to the stirred mixture for over 15 min and stirring was continued for 24 h under N_2 atmosphere. The solvent was removed in vacuo and the residue was extracted with ethyl acetate and water. The organic layer was washed with brine, dried over anhydrous sodium sulfate, and concentrated in vacuo to give **1** as a pure white solid product (4.0 g, 11.4 mmol, 64%). Spectroscopic analysis conformed to previous reports.^{1}H NMR (400 MHz, DMSO-d6) δ 11.77 (s, 1H), 8.20 (s, 1H), 4.38 (s, 2H), 1.42 (s, 9H). ^{13}C NMR (100.6 MHz, DMSO-d6) δ 167.5, 161.5, 151.1, 150.6, 82.5, 68.5, 49.6, 28.1.

tert-Butyl 2-(5-formyluracil-1-yl)acetate (**2a**)

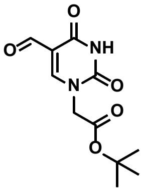

Compound **2a** was prepared according to the procedure previously published.[3]

tert-Butyl 2-(5-(*p*-formylphenyl)uracil-1-yl)acetate (**2b**)

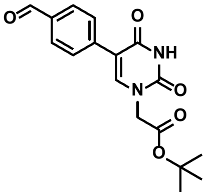

A mixture of 4-formylphenylboronic acid (0.60 g, 1.70 mmol) and K_2CO_3 (0.35 g, 1.5 mmol) in 4:1 THF:water (15 mL) was stirred and degassed with N_2 for 15 min. Pd(dppf)Cl_2 (0.21 g, 0.15 mmol) was added followed by addition of 4-formylphenylboronic acid (0.51 g, 2.0 mmol) to the reaction mixture and heated at 95 °C for 6 h. The reaction mixture was filtered through celite, concentrated in vacuo, and purified by column chromatography using 0-5% methanol in DCM. Yield: 62% (light yellow solid); ^{1}H NMR (400 MHz, DMSO-d6, ppm) *δ:* 11.74 (s, 1H), 10.01 (s, 1H), 8.23 (s, 1H), 7.95 (d, 2H, *J*=8 Hz), 7.85 (d, 2H, *J*=8 Hz), 4.51 (s, 2H), 1.44 (s, 9H). ^{13}C NMR (100.6 MHz, DMSO-d6) *δ* 193.1, 167.5, 162.8, 150.7, 145.8, 139.6, 135.2, 129.9, 128.5, 82.5, 28.2. MS (ESI, +ve mode) calcd. for dimer mass [$(C_{17}H_{18}N_2O_5)_2Na+$]: 683.2324, found: 683.2341.

tert-Butyl 5-(*p*-formylstyryl)uracil-1yl)acetate (**2c**)

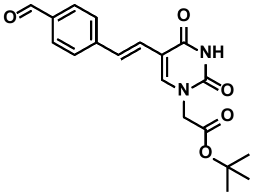

A mixture of **SI-2** (1.5 g, 4.3 mmol) and K_2CO_3 (0.9 g, 6.4 mmol) in 4:1 THF/water (40 mL) was stirred and degassed with N_2 for 15 min. Pd(dppf)Cl_2 (0.5 g, 0.6 mmol) was added followed by addition of **1** (1.1 g, 6.4 mmol) to the reaction mixture and heated at 95 °C for 6 h. The reaction mixture was filtered through celite, concentrated in vacuo and purified by column chromatography using 0-5% methanol in DCM. Yield: 50% (yellow solid); ^{1}H NMR (400 MHz, DMSO-d6, ppm) *δ:* 11.69 (s, 1H), 9.97 (s, 1H), 8.08 (s, 1H), 7.89 (d, 2H, *J*=8 Hz), 7.70 (d, 2H, *J*=8 Hz), 7.53 (d, 1H, *J*=16 Hz), 7.11 (d, 1H, *J*=16 Hz), 4.48 (s, 2H), 1.45 (s, 9H). ^{13}C NMR (100.6 MHz, DMSO-d6) *δ* 192.8, 167.5, 163.0, 150.4, 145.4, 144.0, 135.3, 130.6, 127.0, 126.9, 124.9, 110.3, 82.6, 50.1, 28.2. MS (ESI, +ve mode) calcd. for [$C_{19}H_{20}N_2O_5Na^+$]: 379.1264, found: 379.1298.

4-(Vinylboronic acid pinacol ester)benzaldehyde (**SI-1**)

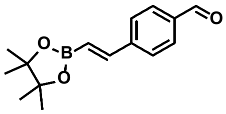

4-bromobenzaldehyde (2.0 g, 10.8 mmol) was dissolved in ACN (50 mL) and triethylamine (3 mL, 21.6 mmol) was added to it. The mixture was degassed with nitrogen for 15 min after which 1,10-phenanthroline and $Pd(OAc)_2$ were added. Vinyl boronic acid pinacol ester was added to the mixture and solution was stirred at 90 °C for 48 h. The reaction mixture was then filtered through celite, diluted with water, and extracted with DCM (3×), dried over anhydrous sodium sulfate, and concentrated in vacuo. Crude was purified by column chromatography using 50-80% DCM in Hexane to yield 2 g (yield: 72%) of light-yellow powder. ^{1}H NMR (400 MHz, DMSO-d6) δ 10.01 (s, 1H), 7.92 (d, 2H, *J*=8 Hz), 7.83 (d, 2H, *J*=8 Hz), 7.41 (d, 1H, *J*=16 Hz), 6.39 (t, 1H), 1.26 (s, 12H). ^{13}C NMR (100.6 MHz, DMSO-d6) δ 193.1, 148.3, 142.9, 136.7, 130.4, 128.2, 83.8. MS (EI, +ve mode) calcd. for [$C_{15}H_{19}BNaO_3^+$]: 281.1319, found: 281.1325.

4-(vinylboronic acid)benzaldehyde (**SI-2**)

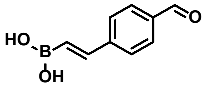

SI-1 (1.0 g, 3.9 mmol) was dissolved in 30 mL (4:1) THF:H_2O, sodium periodate was added to the solution and the mixture was stirred for 30 min. 4.3 mL of 1(M) HCl was then added to the mixture and the solution was stirred for 2 h. The reaction mixture was extracted with DCM (3x), dried over anhydrous sodium sulfate and concentrated in vacuo. The crude was then purified by column chromatography using 1-5% methanol in DCM. Product **2** was obtained as light-yellow solid (0.6 g, 85%). ^{1}H NMR (400 MHz, DMSO-d6) δ 9.99 (s, 1H), 7.92 (d, 2H, *J*=8 Hz), 7.79 (d, 2H, *J*=8 Hz), 7.63 (s, 1H), 7.49 (s, 1H), 7.40 (d, 1H, *J*=16 Hz), 6.98 (d, 1H, *J*=16 Hz). ^{13}C NMR (100.6 MHz, DMSO-d6) δ 192.9, 143.2, 135.7, 133.8, 133.0, 130.5, 127.6.[1]

General method for preparation of **3a, 3b** and **3c.**

2a, **2b** or **2c** (0.9 mmol) was suspended in anhydrous ethanol and malononitrile (2.7 mmol) was added to the mixture. The reaction mixture was heated at 50 °C for 16 h. The reaction mixture was concentrated and purified by column chromatography using 0-5% methanol in DCM.

tert-Butyl (5-dicyanovinyluracil-1-yl)acetate (**3a**)

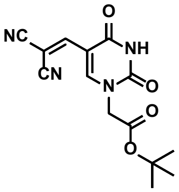

Yield: 96%. ^{1}H NMR (400 MHz, DMSO-d6) δ 9.86 (br s, 1H), 8.59 (s, 1H), 8.03 (s, 1H), 4.55 (s, 2H), 1.53 (s, 9H). ^{13}C NMR (100.6 MHz, DMSO-d6) δ 165.3, 160.8, 150.0, 148.8, 148.4, 113.1, 113.0, 107.5, 84.9, 80.1, 50.6, 28.0. MS (ESI, +ve mode) calcd. for [$C_{11}H_{14}N_2O_5Na^+$]: 325.0907, found: 325.0908.

tert-Butyl 2-(5-(*p*-dicyanovinylphenyl)uracil-1-yl)acetate (**3b**)

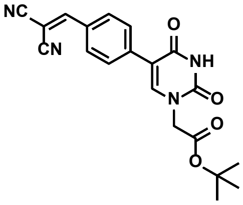

Yield: 76%; ^{1}H NMR (400 MHz, DMSO- d^6) δ 11.76 (s, 1H), 8.52 (s, 1H), 8.27 (s, 1H), 7.98 (d, 2H, J=8 Hz), 7.87 (d, 2H, J=8 Hz), 4.50 (s, 2H), 1.43 (s, 9H). ^{13}C NMR (100.6 MHz, DMSO-d6) δ 167.3, 162.6, 161.2, 150.6, 146.1, 139.6, 131.0, 130.3, 128.5, 114.8, 113.9, 111.3, 82.5, 81.0, 50.0, 28.1; MS (ESI, +ve mode) calcd. for [$C_{20}H_{18}N_4O_4Na^+$]: 401.1226, found: 401.1268.

tert-Butyl 2-(5-(*p*-dicyanovinylstyryl)uracil-1-yl)acetate (**3c**)

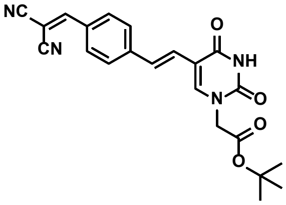

Yield: 74%; ^{1}H NMR (400 MHz, DMSO-d6) δ 11.71 (s, 1H), 8.47 (s, 1H), 8.09 (s, 1H), 7.96 (d, 2H, J=8 Hz), 7.73 (d, 2H, J=8 Hz), 7.54 (d, 1H, J=16 Hz), 7.16 (d, 1H, J=16 Hz), 4.49 (s, 2H), 1.44 (s, 9H); ^{13}C NMR (100.6 MHz, DMSO-d6) δ 167.3, 162.6, 161.2, 150.6, 146.1, 139.6, 131.0, 130.3, 128.5, 114.8, 113.9, 111.3, 82.5, 81.1, 50.0, 28.1; MS (ESI, +ve mode) calcd. for [$C_{22}H_{20}N_4O_4Na^+$]: 427.1377, found: 427.1397.

PHOTOPHYSICAL PROPERTIES

Fluorescence measurements were performed on a Photon Technology International (PTI)QM/TM-40 fluorometer equipped with a 75 W Xenon lamp. Cary 300 spectrophotometer was used to measure the UV-Vis absorption spectra. Molar extinction coefficients were calculated using the Beer-Lambert Law as shown in **equation 1**, where A is absorbance, ε is molar extinction coefficient, c is concentration, ℓ is path length (1 cm) of the light. Quantum yield (φ) in solution phase was measured using rhodamine (0.94 in ethanol) and fluorescein (0.95) as the standard quantum yield reference and using **equation 2**, where φ and φ_{st} are the φ, Grad and $Grad_{st}$ are the gradients obtained by plotting a graph between integrated peak area of fluorescence emission versus absorbance and n and n_{st} are the refractive indexes, of the sample and standard rhodamine respectively. **Table SI-1** gives the complete photophysical characterizations of **3a-c** and **2c** measured for this study. **Figures SI-1**, **SI-2**, **SI-3** and **SI-4** are the fluorescence excitation and emission spectra of **3a-c** and **2c** in different solvents (DMSO, glycerol, methanol, THF and toluene). For all spectra shown, fluorescence intensity increases with increasing concentration.

$A = \varepsilon \times c \times \ell$……………………….**Equation SI-1**

$\varphi = \varphi_{st}\,(Grad/Grad_{st})\,(n^2/n_{st}^2)$……**Equation SI-2**

Table SI-1. Photophysical properties of **DCVSU** (**3c**), **DCVPU** (**3b**), **DCVU** (**3a**).

Compound	Structure	Solvent*	λ_{max} (nm)	λ_{em} (nm)	$\Delta\lambda$ (nm) $\Delta\nu$ (10^3 cm^{-1})	ε (10^4 cm^{-1}M^{-1})	φ	$\varepsilon\times\varphi$ (cm^{-1}M^{-1})
DCVSU (3c)	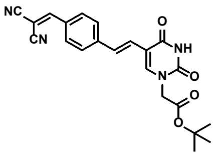	DMSO	411	92	181 (7.4)	4.2	0.21	8700
		Gly	400	573	173 (7.5)	3.7	0.22	8000
		MeOH	395	575	180 (7.9)	2.7	0.09	2390
		Tol	402	496	94 (4.7)	3.1	0.01	310
		THF	404	537	133 (6.1)	2.4	0.05	1190
DCVPU (3b)	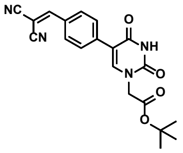	DMSO	374	503	129 (6.9)	1.6	0.004	64
		Gly	370	480	110 (6.2)	2.1	0.011	231
		MeOH	362	482	120 (6.9)	1.5	0.0012	18
		Tol	375	421	46 (2.9)	1.3	0.0004	5
		THF	369	463	94 (5.5)	2.0	0.0011	21
DCVU (3a)	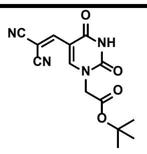	DMSO	350	389	39 (2.6)	Low emission in the range of 1-6 μM		
		Gly	330	398	68 (5.2)	Only tested for fluorescence turn-on at 5 μM		
		MeOH						
		Tol			Undetectable			
		THF						
(**2c**)	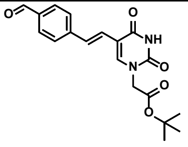	DMSO	354	462	108	3.0	0.03	900
		Gly	351	500	149	1.6	0.16	2560
		MeOH	346	505	159	2.3	0.06	1380
		THF	348	428	80	3.1	0.01	310

*Solvents: DMSO: dimethylsulfoxide; Gly: glycerol; MeOH: methanol; Tol: toluene; THF: tetrahydrofuran.

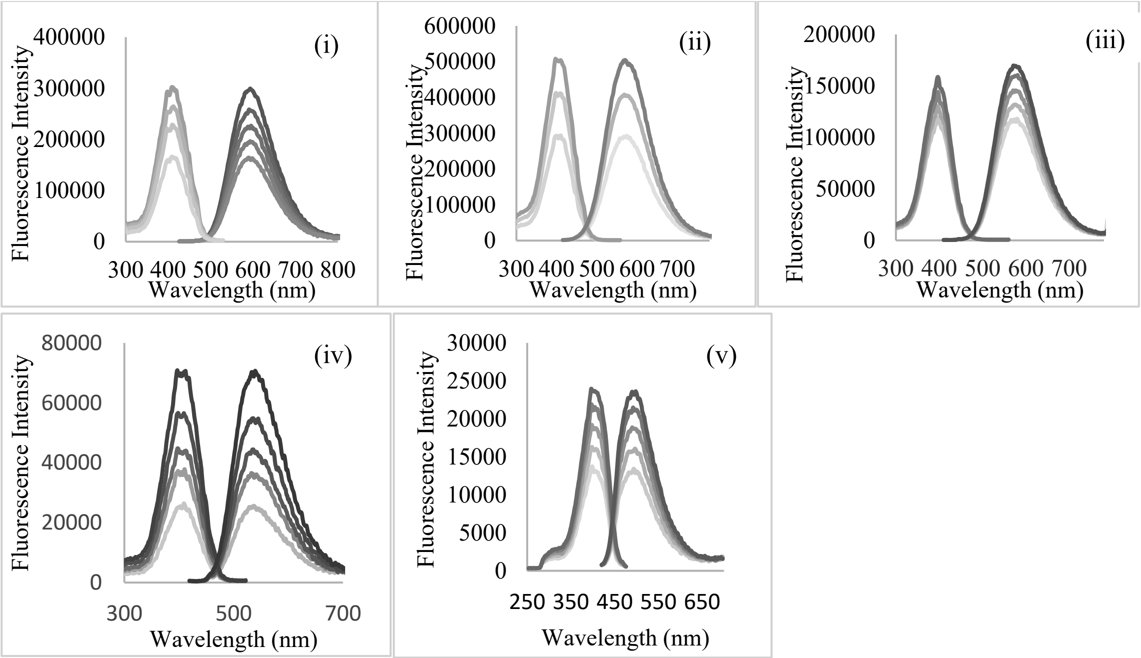

Figure SI-1. Fluorescence excitation and emission spectra of **DCVSU** (**3c**) in (i) DMSO (1-5 μM solutions), (ii) Glycerol* (1-3 μM solutions), (iii) Methanol (1-5 μM solutions), (iv) THF (1-3 μM), (v) Toluene (1-5 μM solutions). *includes 5-10% methanol to ensure complete solubility of **3c**.

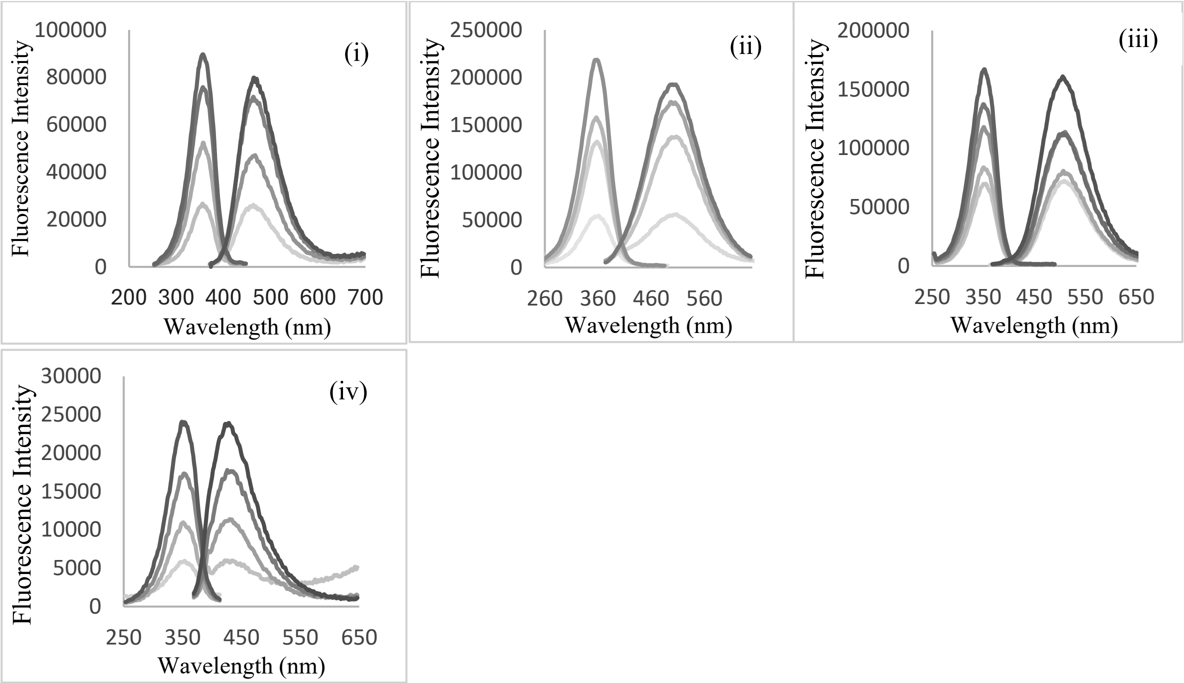

Figure SI-2. Fluorescence excitation and emission spectra of **DCVPU** (**3b**) in (i) DMSO (1-5 μM solutions), (ii) Glycerol* (2-5 μM solutions), (iii) Methanol (1-5 μM solutions), (iv) THF (1-4 μM solutions). *includes 5-10% methanol to ensure complete solubility of **3b**.

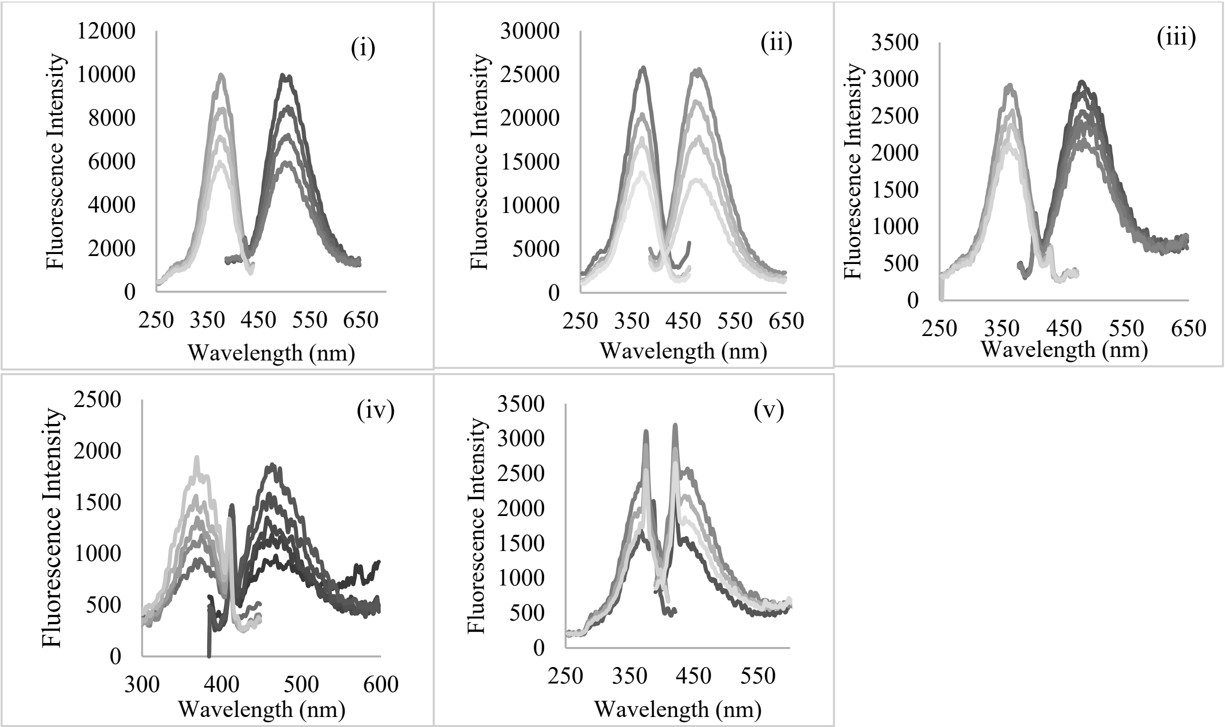

Figure SI-3. Fluorescence excitation and emission spectra of **DCVPU** (**3a**) in (i) DMSO (1-4 μM solutions), (ii) Glycerol* (1-4 μM solutions), (iii) Methanol (1-5 μM solutions), (iv) THF (1-3 μM solutions), (v) Toluene (1-7 μM solutions). *includes 5-10% methanol to ensure complete solubility of **3a**.

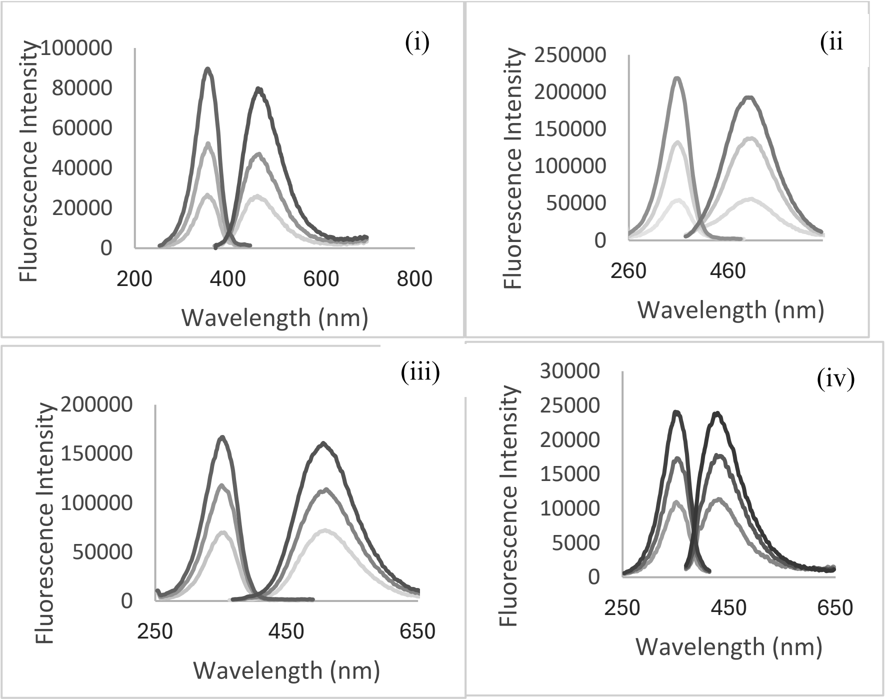

Figure SI-4. Fluorescence excitation and emission spectra of 1, 3 and 5 μM solutions of (**2c**) in (i) DMSO, (ii) Glycerol*, (iii) Methanol, (iv) THF. *included 5-10% methanol to ensure complete solubility of **2c**.

Lippert-Mataga plot for polarity dependent solvatochromism

Stokes shift was measured from the excitation and emission spectra and calculated in wavenumber. Orientation polarizability (Δf) was calculated from literature values of refractive index (n) and dielectric constant (ε) using **eq1b**. Stokes shift was plotted in y-axis and orientation polarizability was plotted in x-axis from which relationship can be drawn between effect on stokes shift with change in dipole moment in the ground state and the excited state of the IFNA using eq1a, also called the Lippert-Mataga equation, where, μ_e and μ_g are excited and ground-state dipole moments, *c* is the velocity of light, *h* is the Planck's constant, and "*a*" is the Onsager cavity radius swept out by the fluorophore.

$$\Delta\bar{v} = \frac{2(\mu_e - \mu_g)^2}{hca^3}\left(\frac{\varepsilon - 1}{2\varepsilon + 1} - \frac{n^2 - 1}{2n^2 + 1}\right) + const \quad \ldots\ldots eq1a$$

$$\Delta f = \left(\frac{\varepsilon - 1}{2\varepsilon + 1} - \frac{n^2 - 1}{2n^2 + 1}\right) \quad \ldots\ldots eq1b$$

				**3c**	**3b**
	(n)	(ε)	Δf	Stokes shift (cm^{-1})	Stokes shift (cm^{-1})
Toluene	1.4969	2.38	0.013	4700	2900
THF	1.4072	7.58	0.21	6100	5500
Methanol	1.3284	32.7	0.309	7900	6900
Glycerol	1.4729	40	0.265	7500	6200
DMSO	1.4793	46.68	0.263	7400	6900

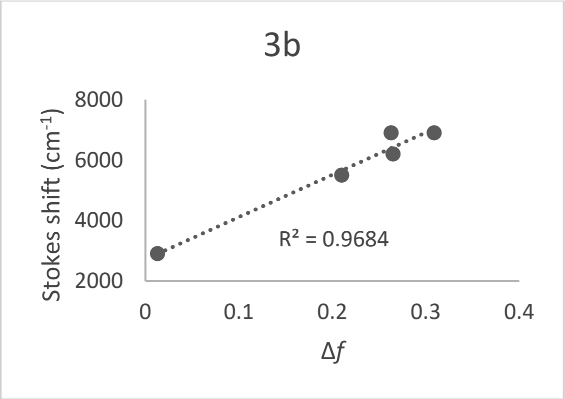

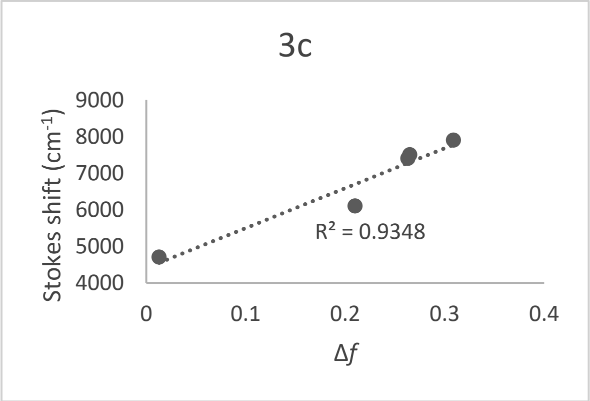

Solvent Polarity Sensitivity Determined by $E_T(30)$ vs. Stokes Shift Plots

				3c	3b
	(n)	(ε)	$E_T(30)$ (kcalmol^{-1})	Stokes shift (cm^{-1})	Stokes shift (cm^{-1})
Toluene	1.4969	2.38	33.9	4700	2900
THF	1.4072	7.58	37.4	6100	5500
Methanol	1.3284	32.7	55.4	7900	6900
Glycerol	1.4729	40	-	7500	6200
DMSO	1.4793	46.68	45.1	7400	6900

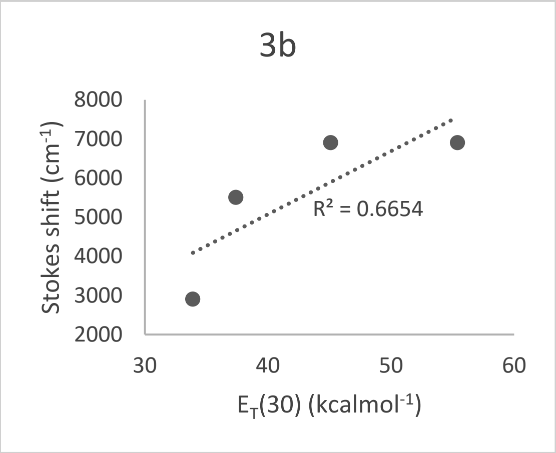

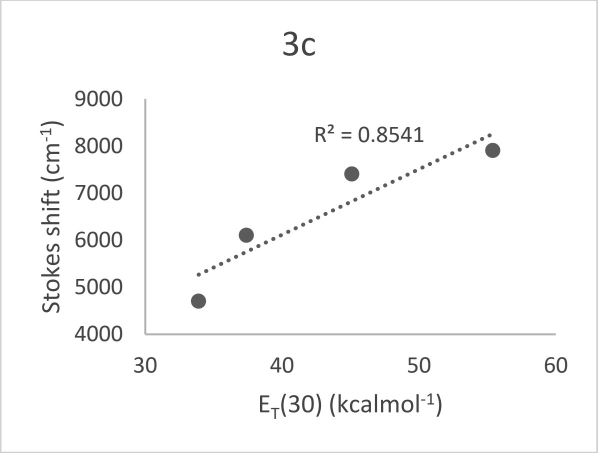

Complementary nucleobase Association studied by ^{1}H NMR

Using ^{1}H NMR spectroscopy, the association of adenine-9-ethyl acetate with dicyanovinyl-based modified uracil derivatives was studied. The host (H), adenine-9-ethyl acetate (0.5 mL of 0.5 mM in $CDCl_3$), was placed in an NMR tube to which 20 mM aliquots of **3a** (guest, G) in $CDCl_3$. The guest solution also contained 0.5 mM host so that the host concentration was constant throughout the experiment. Aliquots (20 μL) of guest solution were added sequentially to the host solution; after addition the NMR tube was vigorously mixed for a brief period using a vortex machine and then ^{1}H NMR spectra were collected. The formation of the complex (H:G) between adenine-9-ethyl acetate and **3a** was observed by downfield shift of the signals of the N^6 and C^2 hydrogens of the host due to hydrogen bonding and pseudo-hydrogen bonding interactions with O^2 and O^4 of modified uracil **3a**, respectively. The variation of the chemical shift (δ) with increasing guest concentration was fit to the Benesi-Hildebrand equation (**equation 3**) to determine association constant, K_a. The curve was fitted in a 1:1 complex formation equation (**equation 4**), the best fit for the curve was obtained. K_a was found to be 155.3±10.5 M^{-1} for N^6H--O H-bonding and 154.7±13.2 M^{-1} for C^2H--O pseudo-hydrogen bonding interactions (**Figure SI-5 a**).

$$1/\Delta\delta = 1/(K_a\ \Delta\delta_{max}[H]_0) + 1/\Delta\ \Delta\delta_{max} \qquad \text{Equation SI-3}$$

$$[HG] = \frac{(Ka[H]0 + Ka[G]0 + 1) - \sqrt{(Ka[H]0 + Ka[G]0) - 2Ka[H]0 + 2Ka[G]0 + 1}}{2Ka} \qquad \text{Equation SI-4}$$

Similar studies were attempted with **3b** and **3c**; however, **3c** was insoluble in $CDCl_3$ and **3b** was not sufficiently soluble (~ 2 mM in $CDCl_3$) to perform the analysis. Interchanging the roles of host and guest were not possible, as adenine-9-ethyl acetate was not completely soluble beyond 5 mM. Although, quantitative analysis was not possible, with the low concentrations of host and guest (**3b**), we observed the downfield shift of N^3H due to interactions with N^1 of adenine-9-ethyl acetate (**Figure SI-6**).[4]

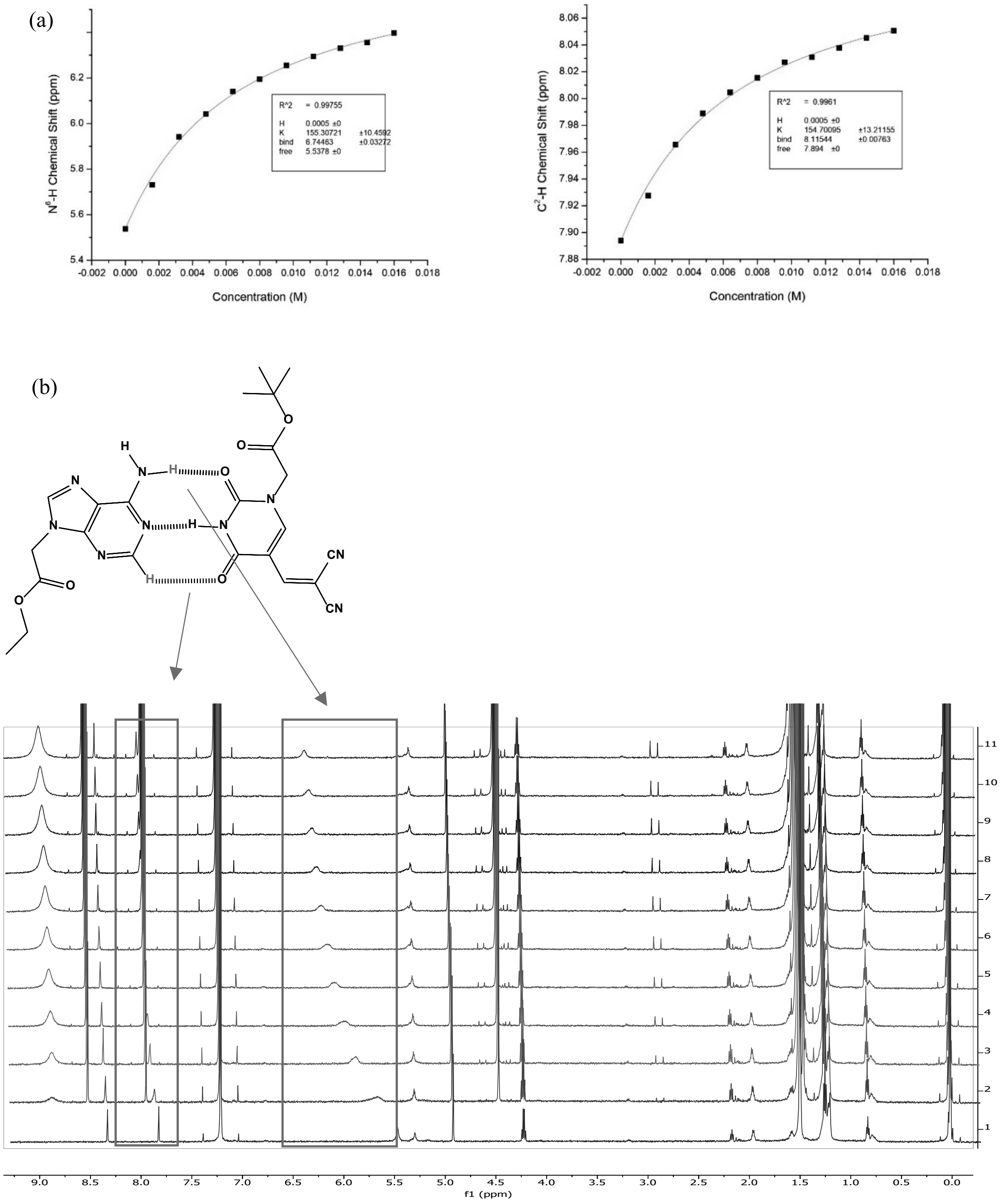

Figure SI-5. a) $N^6\underline{H}$ and $C^2\underline{H}$ chemical shifts of adenine (host) *vs.* concentration of **3a** (guest) plots using non-linear curve fit, b) ^{1}HNMR titration of 0.5 mM adenine-9-ethylacetate (host) with 20 mM **3a** (guest).

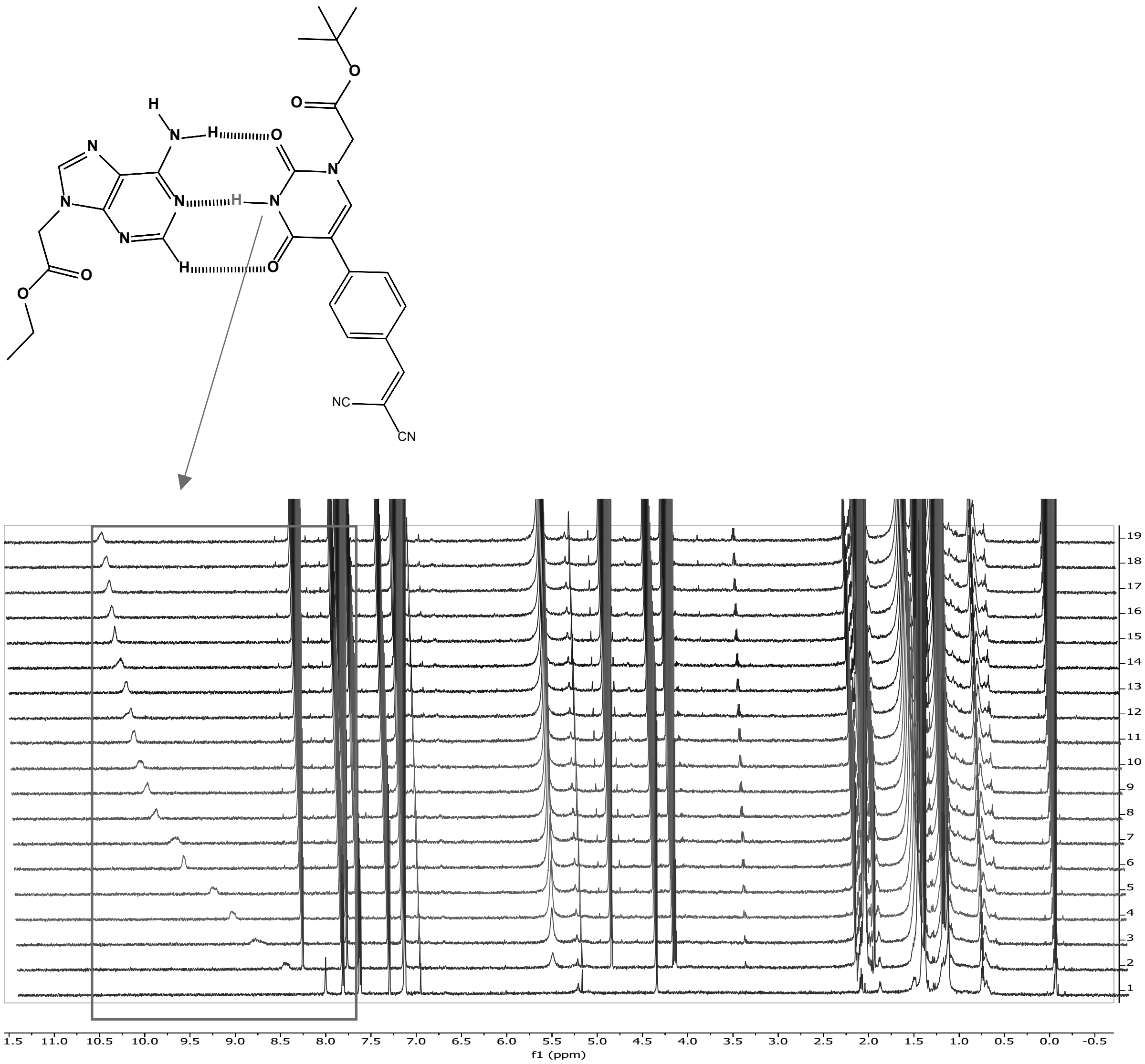

Figure SI-6. ^{1}H NMR titration of 0.5 mM **3b** (host) with 5 mM adenine-9-ethyl acetate (guest), the chemical shift change for N^3-H is highlighted in the box.

NMR spectra

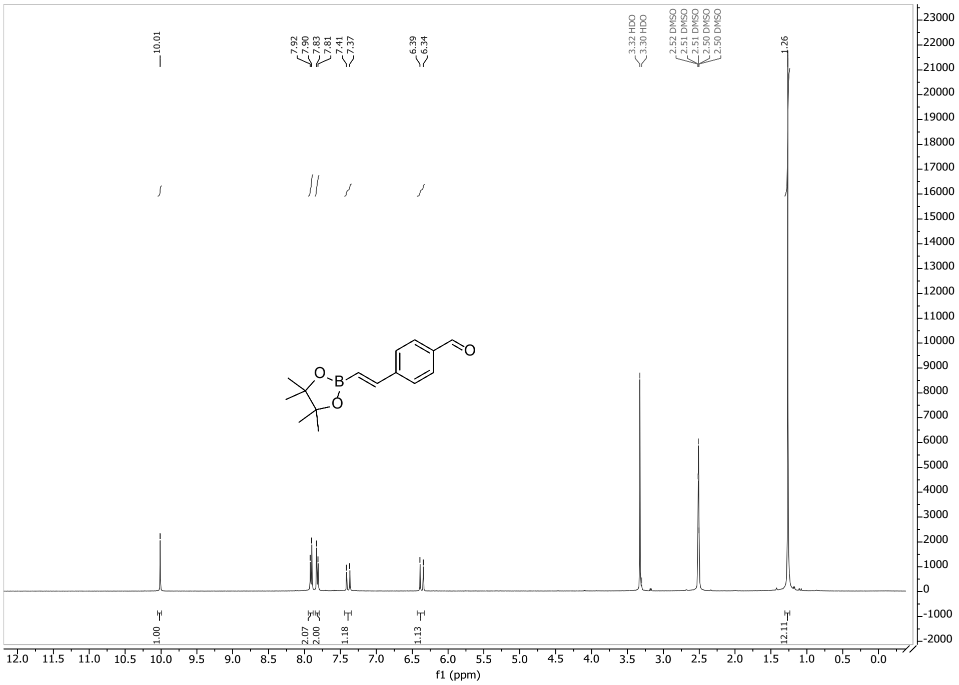

^{1}H NMR of (**SI-1**)

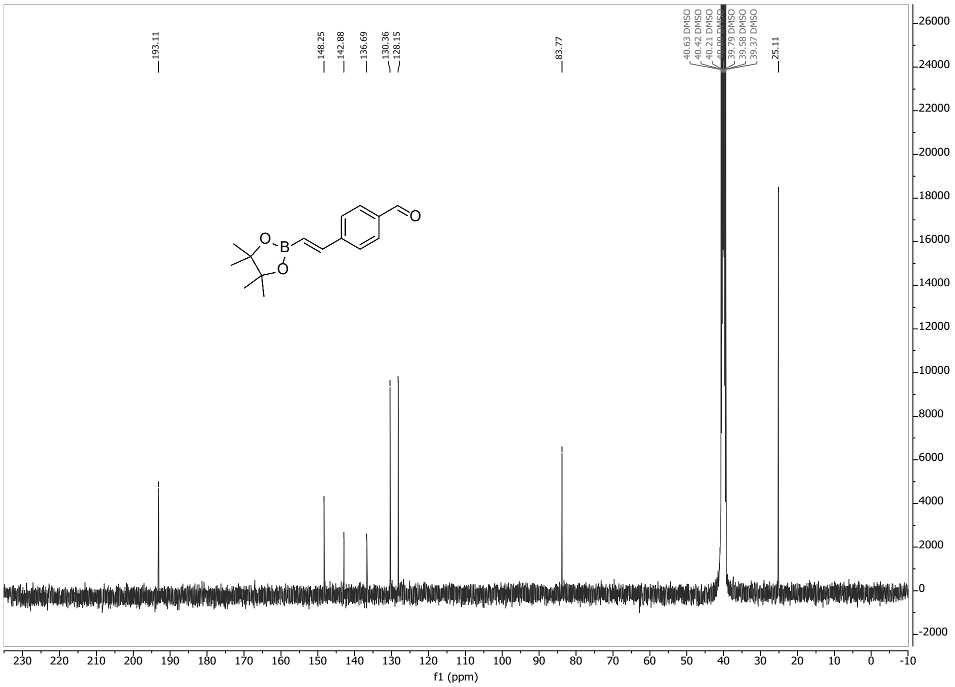

^{13}C NMR of (**SI-1**).

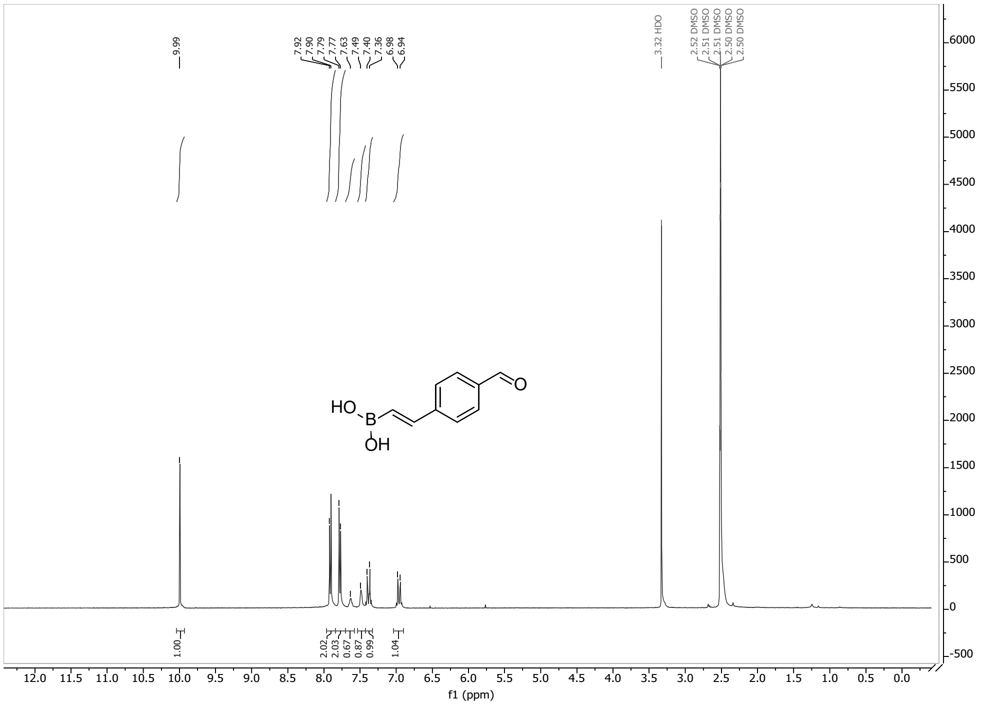

^{1}H NMR of (**SI-2**)

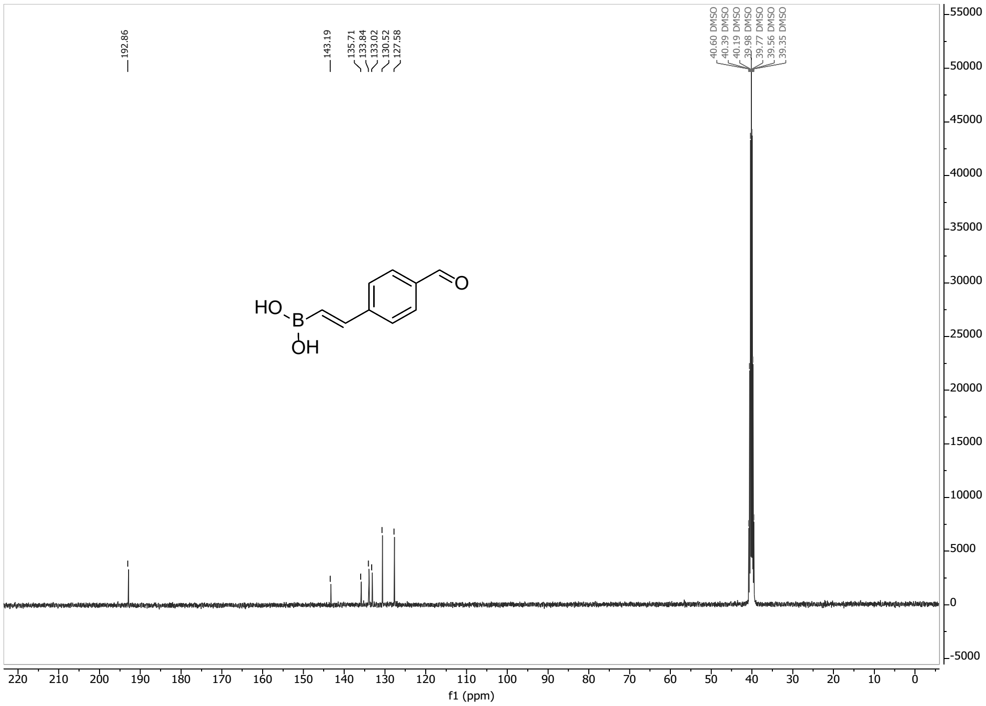

^{13}C NMR of (**SI-2**)

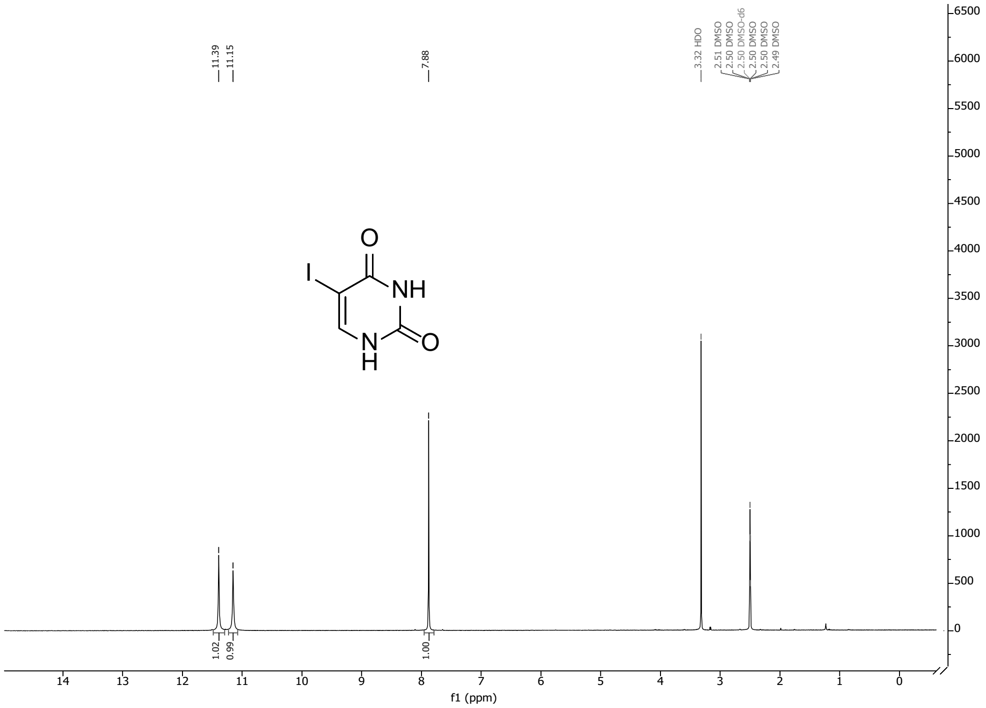

^{1}H NMR of 5-iodouracil

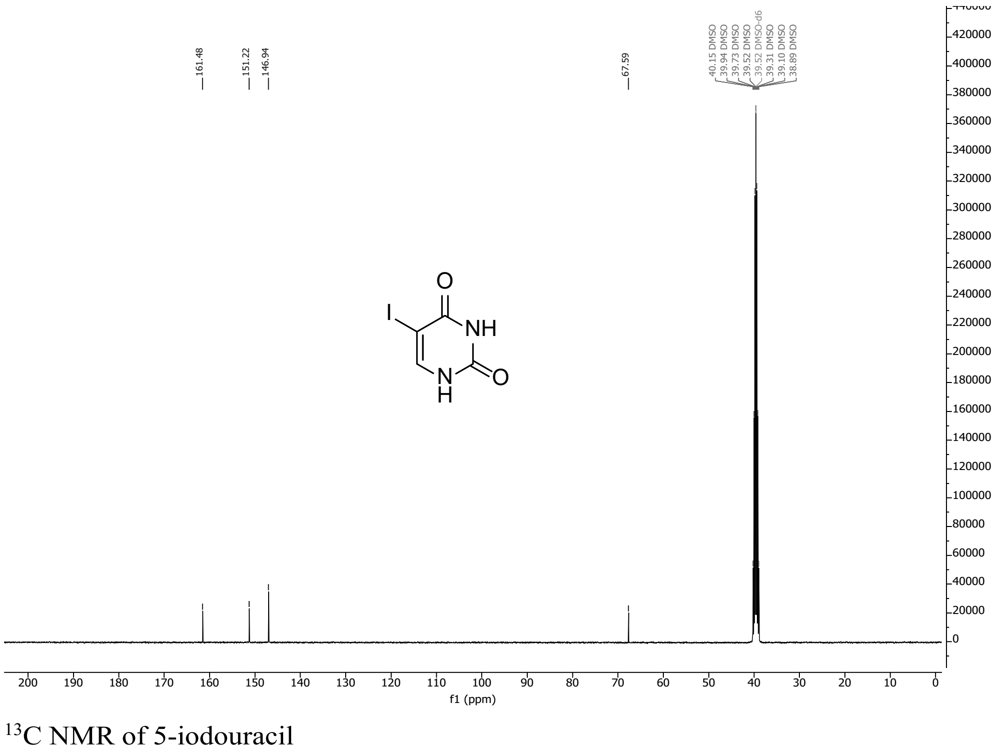

^{13}C NMR of 5-iodouracil

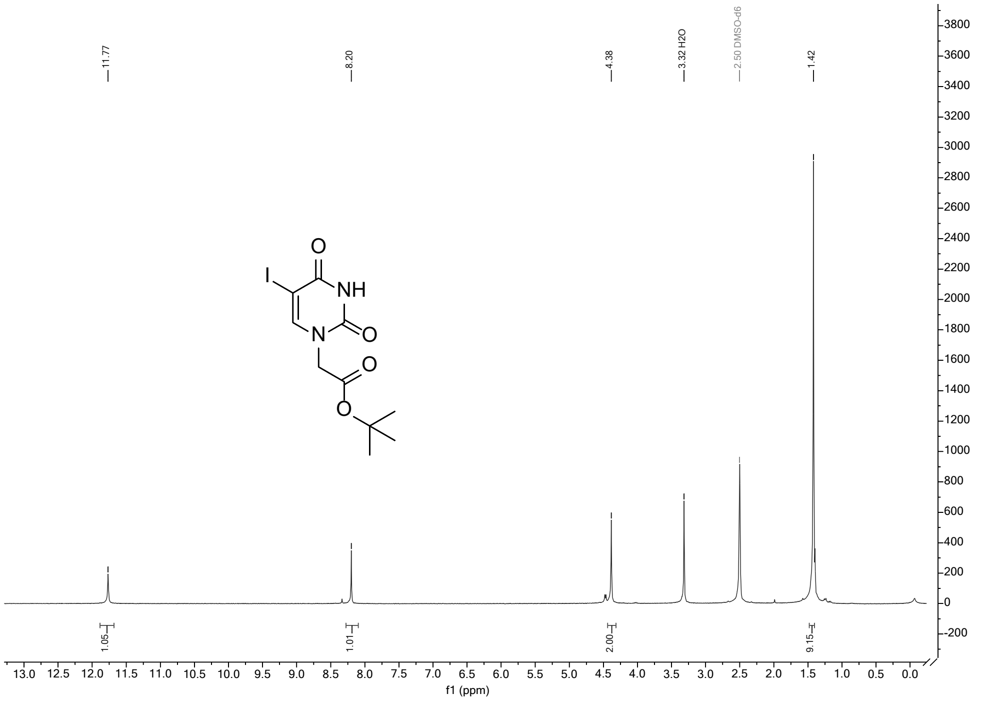

^{1}H NMR of (**1**)

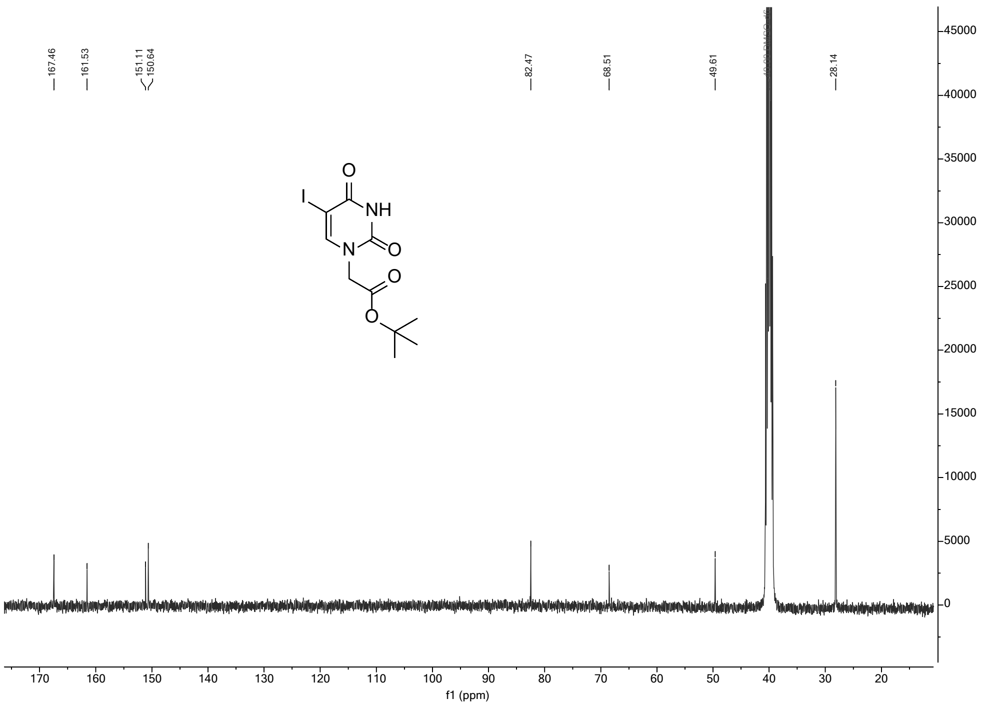

^{13}C NMR of (**1**)

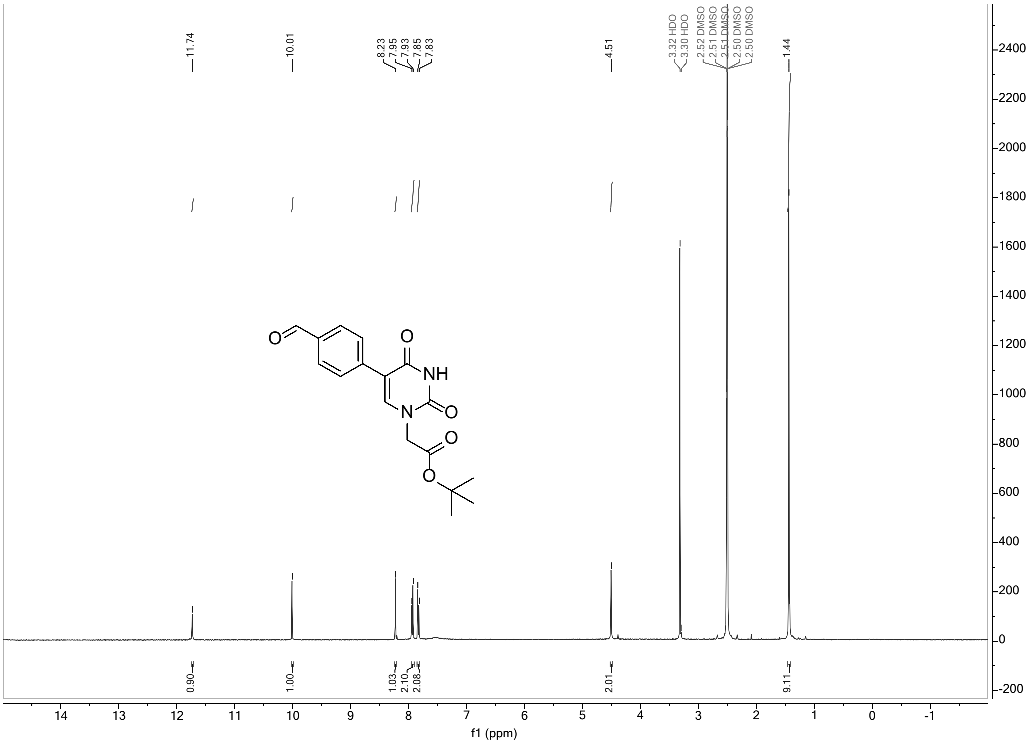

^{1}H NMR of (**2b**)

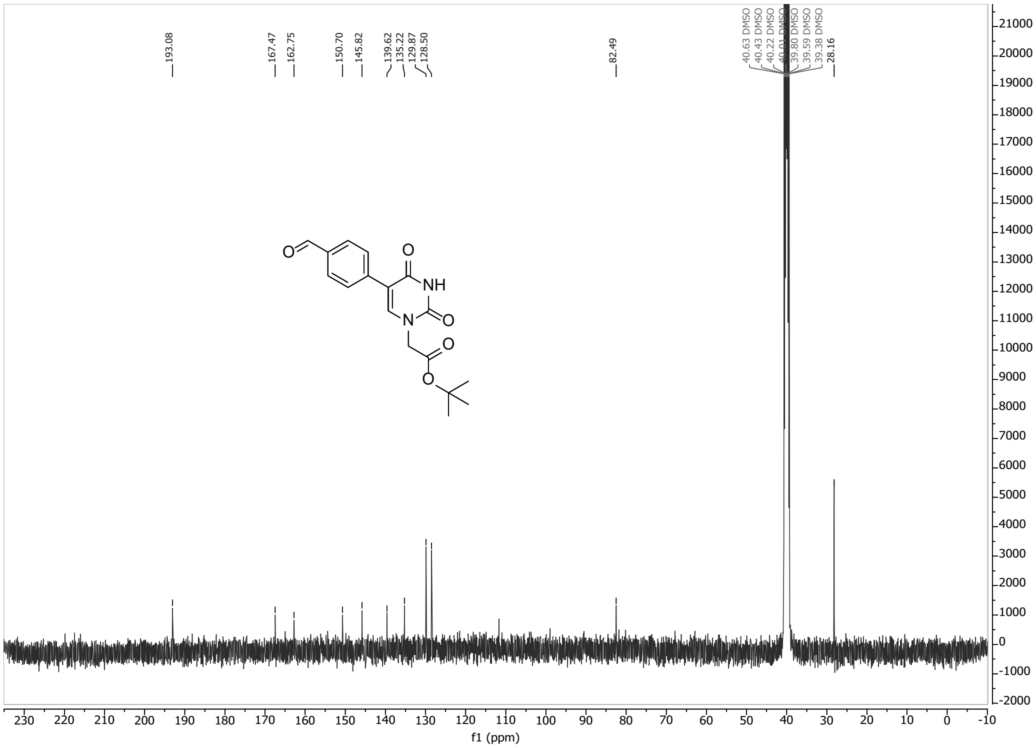

^{13}C NMR of (**2b**)

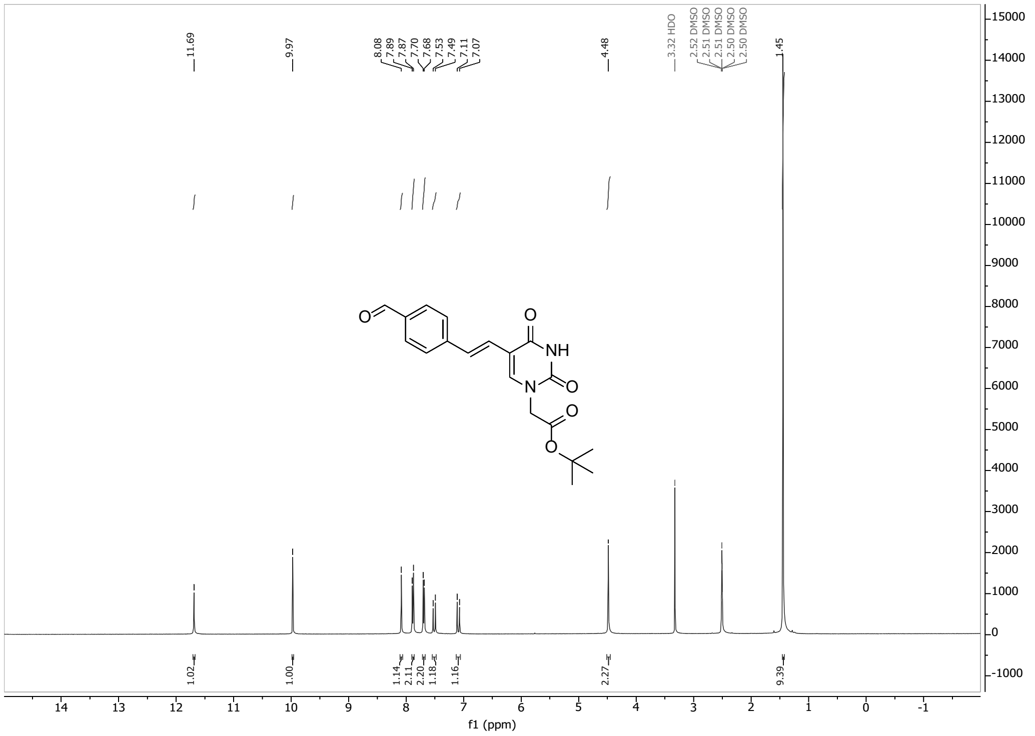

^{1}H NMR of (**2c**)

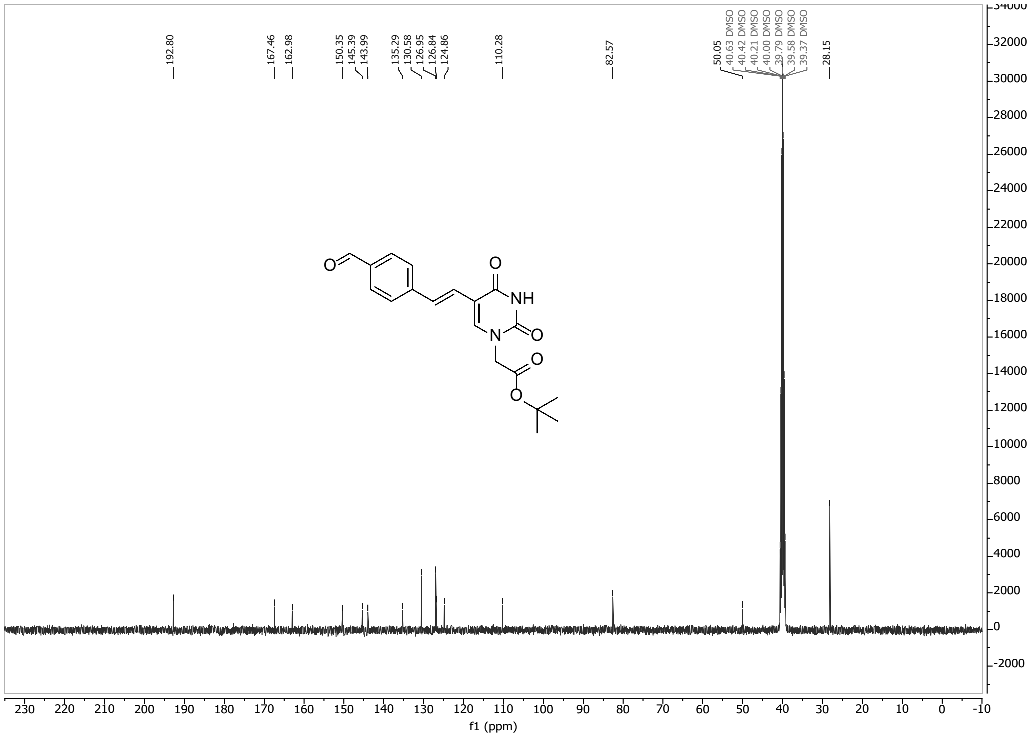

^{13}C NMR of (**2c**)

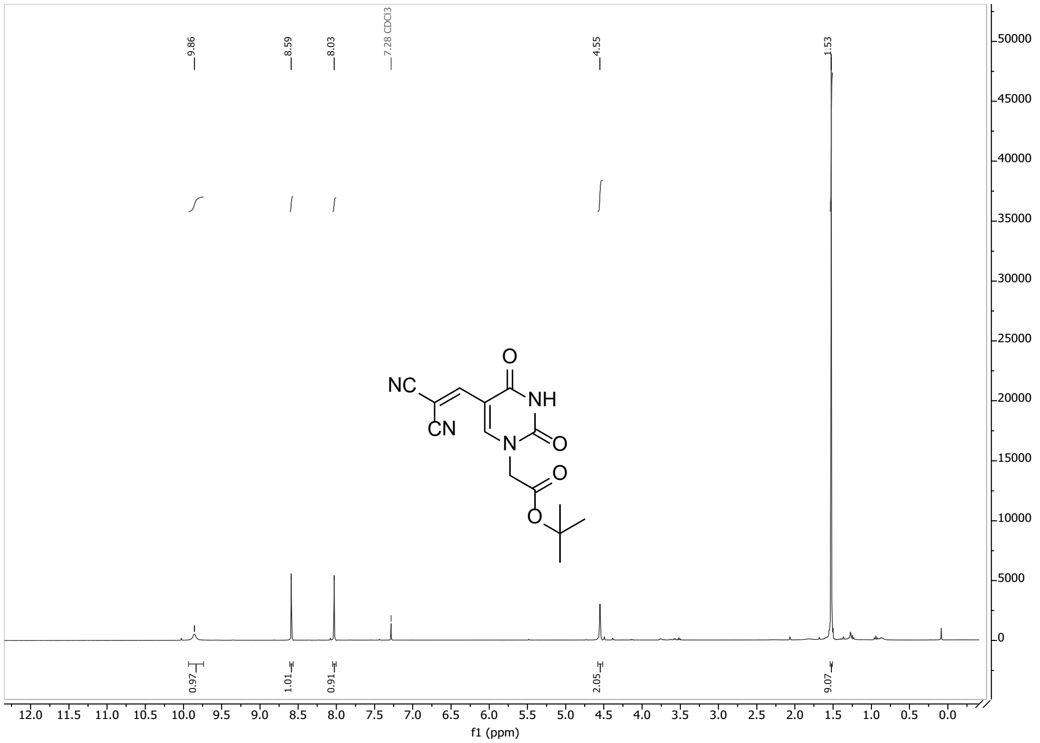

^{1}H NMR of (**3a**)

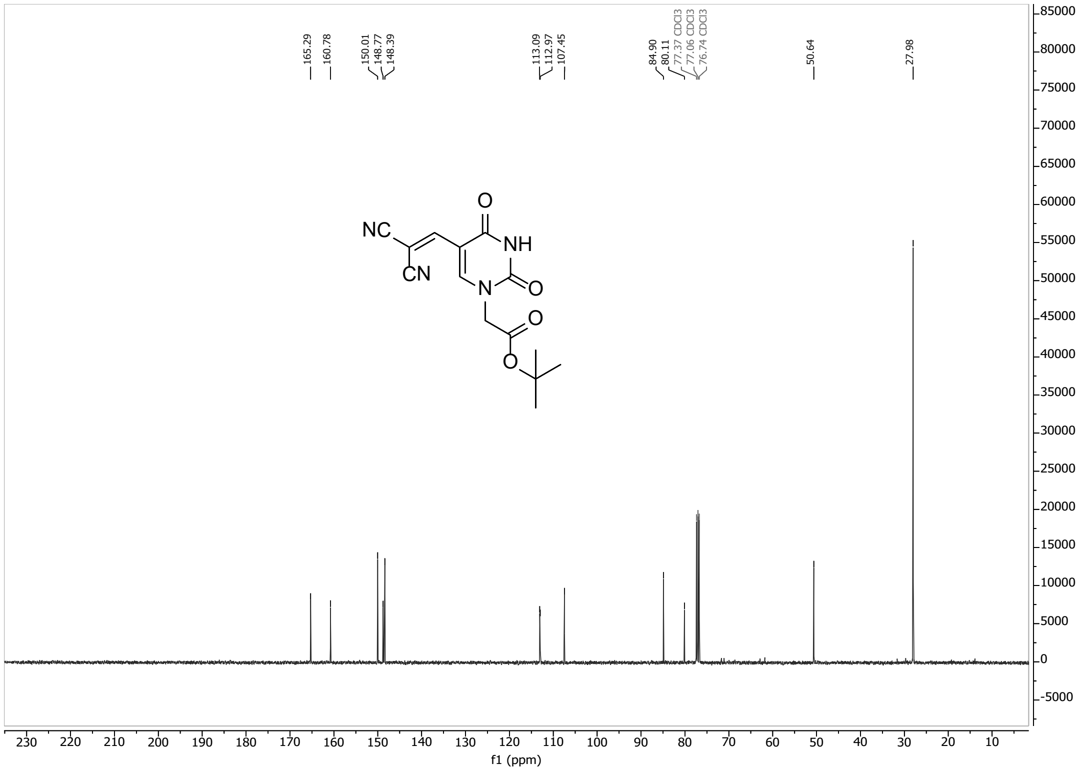

^{13}C NMR of (**3a**)

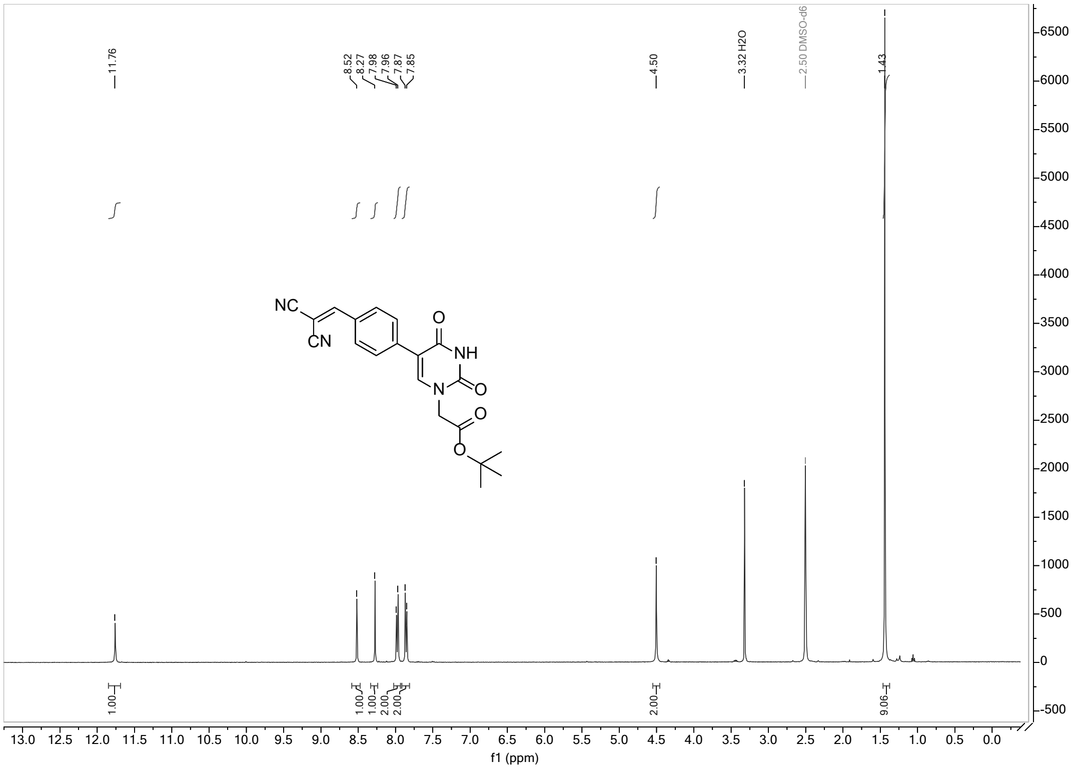

^{1}H NMR of (**3b**)

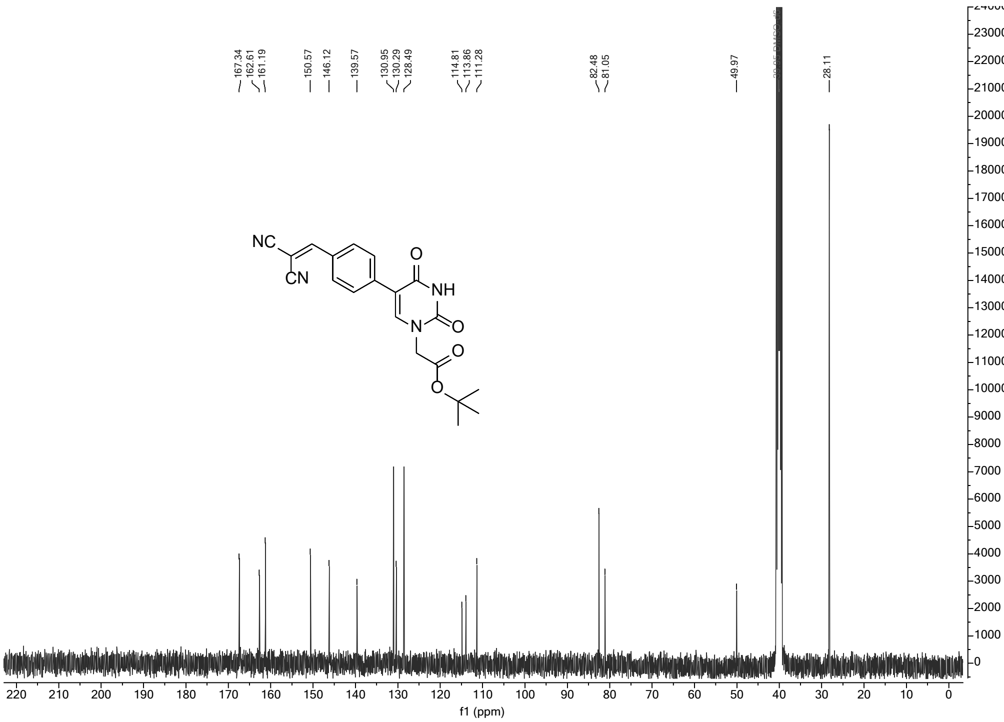

^{13}C NMR of (**3b**)

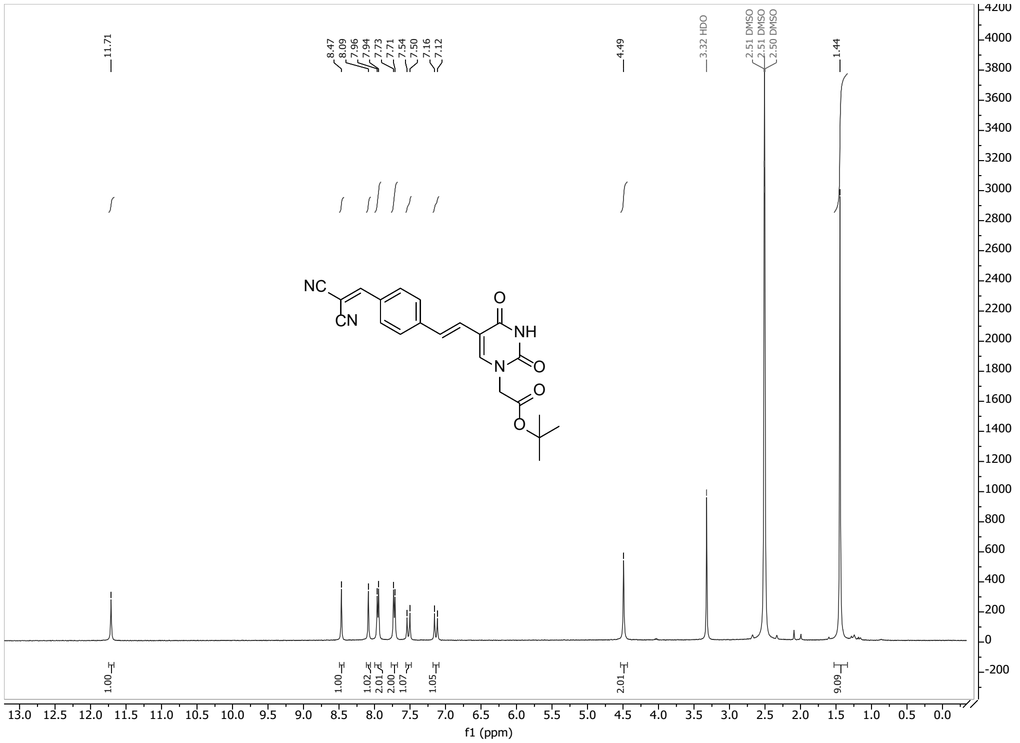

^{1}H NMR of (**3c**)

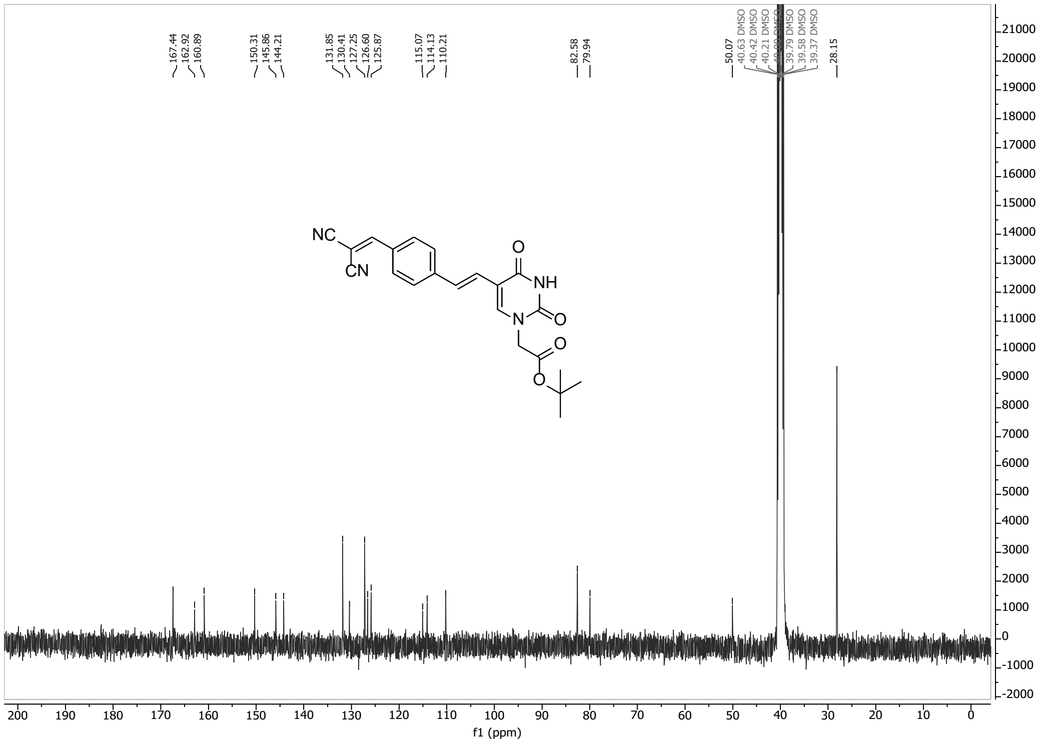

^{13}C NMR of (**3c**)

ESI-MS DATA

SI-1, [$C_{15}H_{19}BNaO_3^+$]: 281.1319, found: 281.1325.

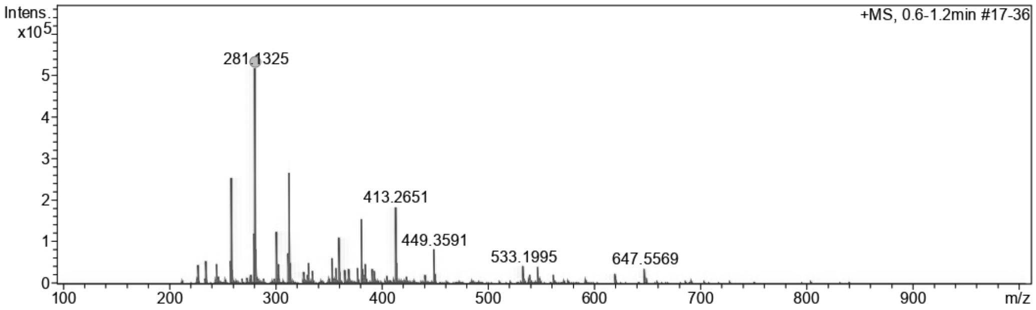

Meas. m/z	#	Ion Formula	m/z	err [ppm]	mSigma	# mSigma	Score	rdb	e⁻ Conf	N-Rule
281.1325	1	C15H19BNaO3	281.1319	-1.0	6.3	1	100.00	6.5	even	ok

2b, dimer mass [$(C_{17}H_{18}N_2O_5)_2Na^+$]: 683.2324, found: 683.2341.

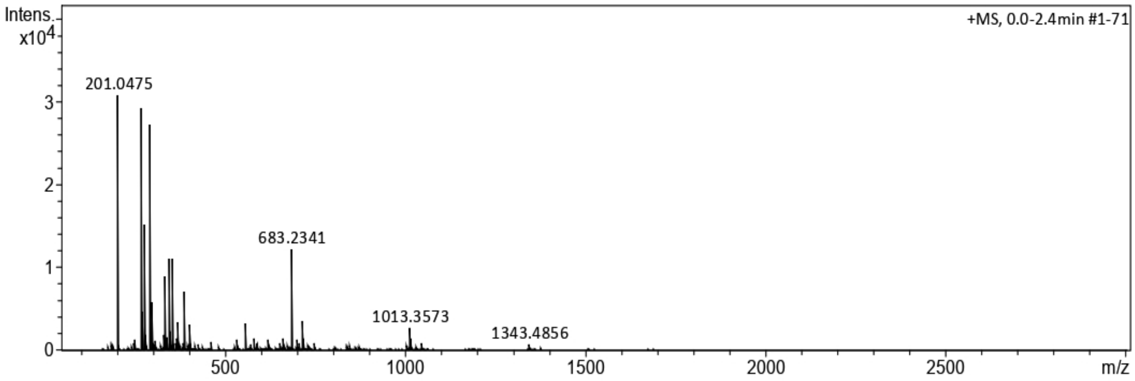

2c, [$C_{19}H_{20}N_2O_5Na^+$]: 379.1264, found: 379.1298, dimer mass [$C_{38}H_{40}N_4O_{10}Na^+$]: 735.2637, found: 735.2668.

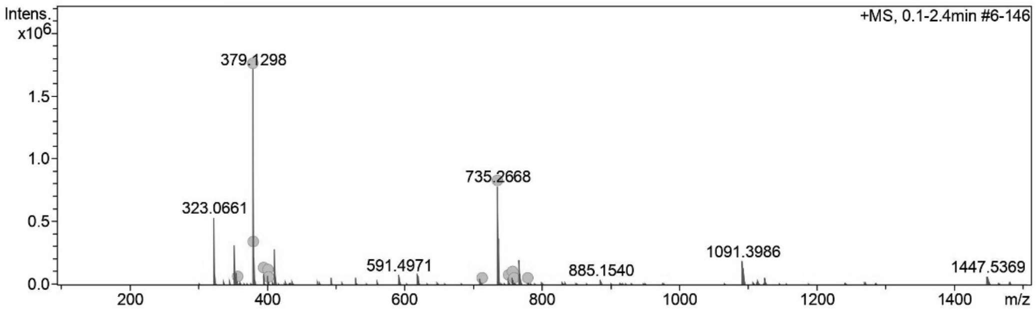

3a, [$C_{11}H_{14}N_2O_5Na^+$]: 325.0907, found: 325.0908

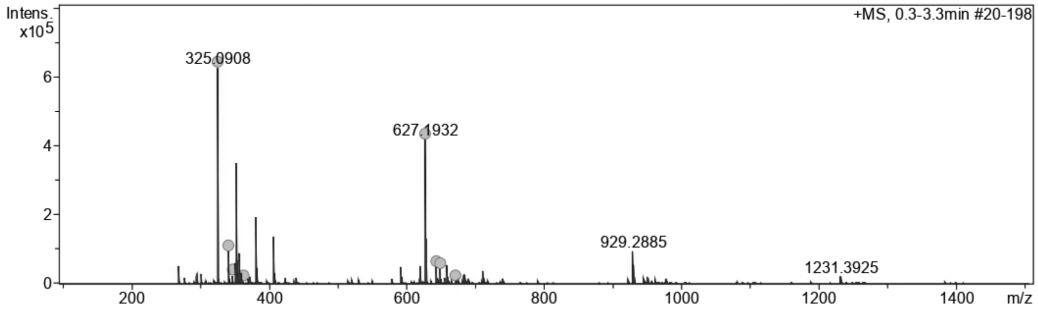

Meas. m/z	#	Ion Formula	m/z	err [ppm]	mSigma	# mSigma	Score	rdb	e⁻ Conf	N-Rule
325.0908	1	C16H13N4O4	325.0931	7.2	22.2	1	100.00	12.5	even	ok
	1	C16H13N4O4	325.0931	7.2	22.2	1	100.00	12.5	even	ok
	1	C14H14N4NaO4	325.0907	-0.2	9.7	1	100.00	9.5	even	ok

3b, [$C_{20}H_{18}N_4O_4Na^+$]: 401.1226, found: 401.1268.

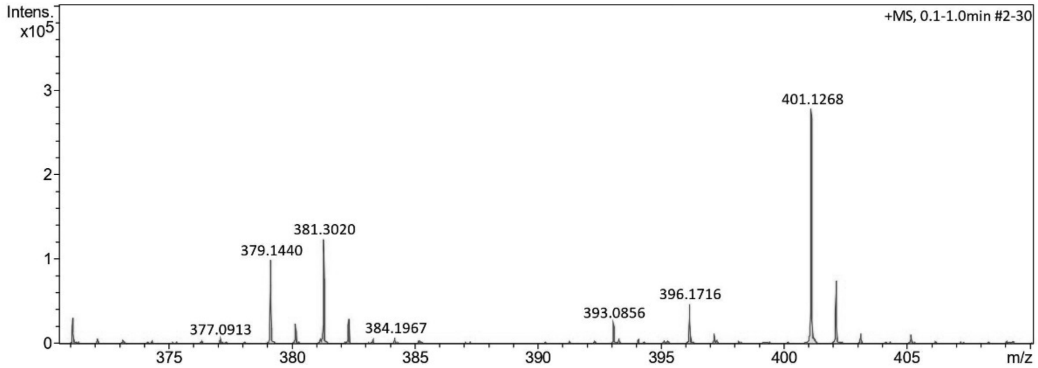

3c, [$C_{22}H_{20}N_4NaO_4^+$]: 427.1377, found: 427.1397.

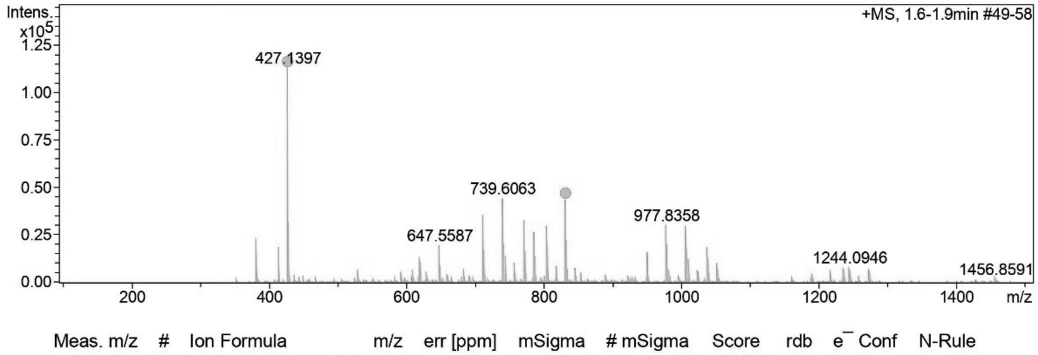

Meas. m/z	#	Ion Formula	m/z	err [ppm]	mSigma	# mSigma	Score	rdb	e⁻ Conf	N-Rule
427.1397	1	C24H19N4O4	427.1401	0.8	0.4	1	100.00	17.5	even	ok
	1	C22H20N4NaO4	427.1377	-4.8	12.4	1	100.00	14.5	even	ok
831.2876	1	C46H39N8O8	831.2885	1.2	13.5	1	100.00	31.5	even	ok
	1	C44H40N8NaO8	831.2861	-1.7	13.8	1	100.00	28.5	even	ok

REFERENCES

1 B. Dang, M. Guo, Peptide tags, compositions and methods for site-specific protein conjugation, PCT/CN2023/130506, 08 November, 2023.

2 R. H. E. Hudson, G. Li and J. Tse, *Tetrahedron Lett*, 2002, **43**, 1381–1386.

3 M. Chowdhury, J. A. Turner, D. Cappello, M. Hajjami and R. H. E. Hudson, *Org Biomol Chem*, 2023, **21**, 9463–9470.

4 P. Thordarson, *Chem Soc Rev*, 2011, **40**, 1305–1323.
